# Ketohexokinase-dependent metabolism of cerebral endogenous fructose in microglia drives diabetes-associated cognitive dysfunction

**DOI:** 10.1038/s12276-023-01112-y

**Published:** 2023-11-01

**Authors:** Yansong Li, Tao Jiang, Mengyu Du, Shuxuan He, Ning Huang, Bo Cheng, Chaoying Yan, Wenxin Tang, Wei Gao, Hongyan Guo, Qiao Li, Qiang Wang

**Affiliations:** 1 Department of Anesthesiology & Center for Brain Science, The First Affiliated Hospital of Xi’an Jiaotong University, 710061 Xi’an, Shaanxi China; 2 Department of Anesthesiology, The Second Affiliated Hospital of Xi’an Jiaotong University, 710004 Xi’an, Shaanxi China; 3 Department of Physiology and Pathophysiology, School of Basic Medical Sciences, Xi’an Jiaotong University Health Science Center, 710061 Xi’an, Shaanxi China; 4 Institute of Neuroscience, Translational Medicine Institute, Xi’an Jiaotong University Health Science Center, 710061 Xi’an, Shaanxi China

**Keywords:** Alzheimer's disease, Diabetes complications

## Abstract

Dementia, as an advanced diabetes-associated cognitive dysfunction (DACD), has become the second leading cause of death among diabetes patients. Given that little guidance is currently available to address the DACD process, it is imperative to understand the underlying mechanisms and screen out specific therapeutic targets. The excessive endogenous fructose produced under high glucose conditions can lead to metabolic syndrome and peripheral organ damage. Although generated by the brain, the role of endogenous fructose in the exacerbation of cognitive dysfunction is still unclear. Here, we performed a comprehensive study on leptin receptor-deficient T2DM mice and their littermate m/m mice and revealed that 24-week-old db/db mice had cognitive dysfunction and excessive endogenous fructose metabolism in the hippocampus by multiomics analysis and further experimental validation. We found that the rate-limiting enzyme of fructose metabolism, ketohexokinase, is primarily localized in microglia. It is upregulated in the hippocampus of db/db mice, which enhances mitochondrial damage and reactive oxygen species production by promoting nicotinamide adenine dinucleotide phosphate oxidase 4 (NOX4) expression and mitochondrial translocation. Inhibiting fructose metabolism via ketohexokinase depletion reduces microglial activation, leading to the restoration of mitochondrial homeostasis, recovery of structural synaptic plasticity, improvement of CA1 pyramidal neuron electrophysiology and alleviation of cognitive dysfunction. Our findings demonstrated that enhanced endogenous fructose metabolism in microglia plays a dominant role in diabetes-associated cognitive dysfunction and could become a potential target for DACD.

## Fructose metabolism in microglia: a new player in diabetes-linked cognitive dysfunction

Researchers found that the rate-limiting enzyme of fructose metabolism, ketohexokinase (KHK), is primarily localized in microglia and upregulated in the hippocampus of diabetic mice, leading to mitochondrial damage and reactive oxygen species production. Inhibiting fructose metabolism via KHK depletion reduces microglia activation, restores mitochondrial homeostasis, improves structural synaptic plasticity, and alleviates cognitive dysfunction. This study demonstrates the dominant role of enhanced endogenous fructose metabolism in microglia in the process of diabetes-associated cognitive dysfunction and suggests KHK as a potential therapeutic target for this condition.

## Introduction

The population of people with diabetes is estimated to be 642 million by 2040^1^. Approximately 37.3% of those over 65 years old suffer from diabetes-associated cognitive dysfunction (DACD), which is divided into mild cognitive impairment and dementia^2^. Dementia accounts for 16% of all deaths in diabetic patients and has become the second leading cause of death in diabetes^3^. Given that little guidance is currently available to address the DACD process, it is imperative to understand the underlying mechanisms and screen out specific therapeutic targets.

Diabetes is characterized by a chronic state of hyperglycemia, which causes up to 30% of glucose to be converted into fructose by the polyol pathway^4^. In this way, glucose is first reduced to sorbitol by aldose reductase (AR) and subsequently oxidized to fructose by sorbitol dehydrogenase (SDH). Although fructose has a role in survival processes, such as the storage of fat and glycogen, the development of insulin resistance, and an increase in sodium reabsorption^5^, excessive fructose consumption has been associated with obesity, diabetes, nonalcoholic fatty liver disease (NAFLD), kidney diseases and cognitive dysfunction^4,6–8^. Fructose could be phosphorylated by ketohexokinase (KHK), a specific enzyme for fructose metabolism. Unlike hexokinase, KHK has no negative feedback, and neither signals of cellular energy sufficiency nor immediate products inhibit its activity. Therefore, unrestricted KHK activity results in the stepwise degradation of ATP to uric acid^8^. The latter can induce the expression of nicotinamide adenine dinucleotide phosphate oxidase (NADPH oxidase, NOXs)^9^. Given that the sole function of NOXs is to generate reactive oxygen species (ROS), the metabolism of excess endogenous fructose in the liver and kidney causes the accumulation of ROS, leading to the development of metabolic syndrome and renal injury^10,11^. Among seven known NOX subtypes, NOX1-5 and dual oxidase (DUOX) 1/2, NADPH oxidase 4 (NOX4) is one of the most extensively distributed isoforms in the central nervous system and has been characterized in different diseases, such as stroke, trauma, tumors and neurodegenerative diseases^12–14^. It is overexpressed in activated microglia and can promote NLRP3 inflammasome activation in microglia^15,16^. Thus, deletion of NOX4 or inhibition of its expression can improve neurological outcomes^14^.

Emerging evidence has demonstrated that the brain not only produces fructose from glucose under hyperglycemic conditions^1,17^ but also metabolizes fructose by KHK^18^. The activity of KHK and AR was 5–10 times, with rates of fructose oxidation 15-150 times higher in the hippocampus than in the liver^19^. Although cerebrospinal fluid fructose was moderately inversely correlated with cognitive function and the number of myelinated fibers^20,21^, the detailed mechanism of this interplay remains obscure. Combined with the compelling evidence that glucose transporter 5 (GLUT5), the major fructose transporter, is predominantly expressed in microglia^22^, we asked whether the metabolism of endogenous fructose is mainly mediated by microglia and is responsible for DACD.

Here, we performed a comprehensive study on leptin receptor-deficient type 2 diabetes mellitus (db/db) mice and their littermate m/m mice and revealed that 24-week-old db/db mice had cognitive dysfunction and excessive endogenous fructose metabolism in the hippocampus by multiomics analysis and further experimental validation. In addition to microglia being primarily responsible for endogenous fructose metabolism in the brain, we also demonstrated that activation of KHK in microglia promoted NOX4 mitochondrial translocation, leading to the overproduction of ROS. Notably, *Khk* knockdown suppressed the effect above and consequently improved synaptic function and alleviated cognitive dysfunction. Our findings uncovered the role of endogenous fructose metabolism in microglia in DACD pathogenesis.

## Materials and Methods

### Animals

Male homozygous leptin receptor-deficient T2DM (Lepr db/Lepr db, db/db) mice and their littermate m/m (m/m) mice were purchased from GemPharmatech Co., Ltd. (Nanjing, China). C57BL/6J-Khk^emlcyagen^ (*Khk* knockout, *Khk* KO) mice were generated by the CRISPR/Cas9 method (Cyagen Biosciences (Suzhou) Inc., Jiangsu, China). All of the mice were housed in a pathogen-free, temperature-controlled environment with a temperature of 22 ± 2 °C, relative humidity of 60 ± 70%, and a light/dark cycle of 12/12 h and fed ad libitum in a standard laboratory. All animals were allowed to acclimate in the animal facility for at least one week prior to being handled. The body weight and water intake of the mice were monitored every 2 days. All selected mice were randomly divided into specified groups. All animal studies (including the mouse euthanasia procedure) were approved by the Animal Care Institution of Xi’an Jiaotong University and conducted according to the ARRIVE guidelines (NO. 2019-060).

### Morris water maze (MWM)

The Morris water maze was performed as previously described^23^. The details are included in the Supplementary material.

### RNA-seq

RNA-seq was performed on 5 biological replicates for db/db and m/m mice and 6 biological replicates for the BV2 cell, BV2 cell + fructose and BV2 cell + fructose +*Khk*-siRNA groups. Total RNA was isolated, purified, and quantified. Library preparation and paired-end sequencing were performed. After cleaning reads and mapping them to the genome of Homo sapiens, FPKM values were calculated. The fold change of differentially expressed genes was > 1.2 or < 0.83, and a *P* value < 0.05 was considered statistically significant. The details are described in the Supplementary material.

### TMT proteomics

Briefly, five separate biological replicates were performed for both db/db and m/m mice to quantify protein expression. TMT-labeled peptides were fractionated by RP chromatography. LC-MS/MS analysis was performed on a Q Exactive Plus mass spectrometer (Thermo Fisher Scientific) coupled to Easy nLC (Thermo Fisher Scientific). MS/MS raw files were processed using the MASCOT engine (Matrix Science, London, UK; version 2.6) embedded into Proteome Discoverer 2.2 and searched against Uniprot_MusMusculus_17027_20200226, downloaded at http://www.uniprot.org. Proteins with a fold change > 1.2 or < 0.83 and a p value (Student’s *t* test) < 0.05 were considered to be differentially expressed proteins. The details are described in the Supplementary material.

### Widely targeted metabolomics

Six separate biological replicates were performed for both db/db and m/m mice. The sample extracts were analyzed using an LC‒ESI‒MS/MS system (UPLC, Shim-pack UFLC SHIMADZU CBM A system, https://www.shimadzu.com/; MS, QTRAP® System, https://sciex.com/). Subsequent analyses were performed using MetaboAnalyst 5.0 (https://www.metaboanalyst.ca/home.xhtml). Differentially expressed metabolites were identified by fold change > 1.2 or < 0.83, VIP > 1, and *P* value (Student’s *t* test) < 0.05. The details are described in the Supplementary material.

### Bioinformatics and statistics for OMICs data

The comprehensive analysis of transcriptomics and metabolomics datasets and enrichment analysis of metabolomics data were performed using the “joint pathway analysis” function of MetaboAnalyst 5.0 (https://www.metaboanalyst.ca/home.xhtml) as previously described^24^. The hypergeometric test was used for the enrichment analysis, as the topology was measured with “degree centrality”. Volcano plot, Venn diagram, enrichment analysis, gene set enrichment analysis, and correlation analysis of DEGs were performed by OmicStudio tools at https://www.omicstudio.cn/tool. Single-cell RNA-Seq data (GSE201644) from the GEO database (http://www.ncbi.nlm.nih.gov/geo) were acquired and analyzed using the R package Seurat (4.3.0)^25^. The details are described in the Supplementary material.

### Viral vectors and infections

Recombinant adeno-associated virus (rAAV2/6 m) expressing U6-driven shRNA and CMV-driven EGFP (rAAV-U6-shRNA-CMV-EGFP-pA) was used. The scrambled shRNA sequence was CCTAAGGTTAAGTCGCCCTCG. The *Khk* shRNA sequence was GCAGCGGATAGAGGAGCACAA, which targeted mouse *Khk* (GenBank: NM_001310524.1). All AAVs were from BrainVTA Wuhan China. After being anesthetized, mice were injected with 500 nl AAV at the bilateral hippocampus of the brain (AP = 1.5 mm posterior to bregma, ML = 1.0 mm lateral to bregma, DV = 1.55 mm below the skull surface). The details are described in the Supplementary material.

### Western blotting

The details are described in the Supplementary material.

### Immunofluorescence staining and analysis

The details are described in the Supplementary material.

### Serum insulin measurement

Blood samples from mice fasted for 6 h were quantitatively measured by a Mouse INS (Insulin) ELISA Kit (xl-Em0483; Xinle, Shanghai, China) according to the manufacturer’s instructions. Blood glucose was measured using a glucometer (Yuyue, Jiangsu, China). The homeostasis model assessment of insulin resistance (HOMA-IR) was calculated as fasting insulin concentration (mU/L) × fasting glucose concentration (mmol/L)/22.5.

### Biochemical detections

The MDA (Beyotime Biotechnology, Shanghai, CN), 8-OHDG (Preferred Biotechnology, Shanghai, CN), ROS (Beibo, Shanghai, CN), NT (Preferred Biotechnology, Shanghai, CN), ATP (Beyotime Biotechnology, Shanghai, CN), ROS (Beyotime Institute of Biotechnology), fructose (Nanjing Jiancheng Bioengineering Institute, Nanjing, CN) and sorbitol (Solarbio, Beijing, CN) levels were measured using specific kits in accordance with the instructions.

### Transmission electron microscopy examination

Transmission electron microscopy was performed as described previously^23^. The details are described in the Supplementary material.

### Golgi staining and analysis

Golgi staining was performed as previously described with minor modifications^26^. The details are described in the Supplementary material.

### Brain slice patch-clamp recording

#### Brain slice preparation

Slice preparation was performed as described previously^26^. The mice were anesthetized with isoflurane and transcardially perfused. They were rapidly decapitated, and their brains were removed quickly and placed in the cutting solution. Hippocampal slices of 300 µm thickness were prepared and left at room temperature for recording. Standard whole-cell patch clamp recordings were performed in hippocampal CA1 pyramidal neurons. Data were acquired using the MultiClamp 700B amplifier (Molecular Devices, USA) and the 1550 A digitizer (Molecular Devices, USA). The details are described in the Supplementary material.

#### Electrophysiological recording

For spontaneous excitatory postsynaptic current (sEPSC) recording, pyramidal neurons were held at -70 mV in the presence of 100 µM picrotoxin recording solution. For miniature excitatory postsynaptic current (mEPSC) recording, pyramidal neurons were held at -70 mV in the presence of 100 µM picrotoxin and 1 µM TTX. The frequency and amplitude of spontaneous/miniature EPSCs (s/mEPSCs) were analyzed and visually confirmed by Mini60 (MiniAnalysis, Synaptosoft, Leonia, USA). To obtain a high signal-to-noise ratio and accurately determine the s/mEPSC amplitude, only events > 10 pA were accepted for analysis^27^.

To measure the intrinsic excitability (action potential) of neurons, the current clamp mode was used, and pulsed depolarization currents with increasing amplitude were injected at steps of 50 pA. Furthermore, 20 µM CNQX, 50 µM DL-AP5 and 100 µM picrotoxin were added to the perfusion buffer to block excitability and inhibitory neurotransmission.

#### Primary cells and cell line culture and treatment

The details are described in the Supplementary material.

### Statistical analysis

Statistical tests were performed with GraphPad Prism software (GraphPad Software, version 7.0). Continuous data were tested for normal distribution and analyzed by one-way ANOVA (followed by Tukey’s multiple comparisons tests) and Student’s *t* test or Kruskal‒Wallis (followed by Dunn’s multiple comparisons tests) and Mann‒Whitney test. Data are expressed as the mean ± SEM. Two-way ANOVA was applied to analyze the MWM and neural dendritic complexity. *p* < 0.05 was considered statistically significant.

## Results

### Boosted endogenous fructose production and metabolism in the hippocampus of db/db mice

**Fig. 1:**
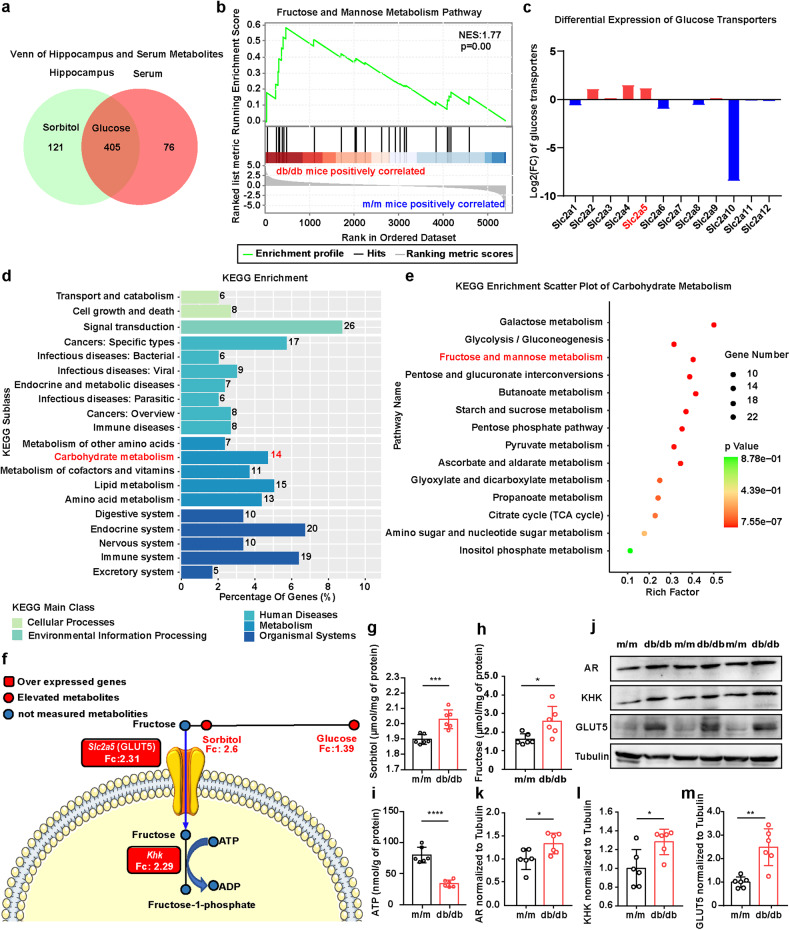
Alterations in fructose production and metabolism in the hippocampus of diabetic mice. **a** Venn diagram of significantly differential metabolites in the serum and hippocampus between db/db and m/m mice (*n* = 6). **b** The alteration of the “fructose and mannose metabolism” pathway in the proteomics of db/db vs. m/m mice via GSEA (NES = 1.77, *P* = 0.0038) (*n* = 5). **c** Fold changes of glucose transporters in RNA-seq analysis of db/db vs. m/m mice (*n* = 5). Red indicates a significant increase in gene expression levels, while blue indicates significantly lower gene expression levels. **d** KEGG enrichment of DEGs related to significantly differential metabolites (*P* < 0.05) (*n* = 5). **e** Fourteen KEGG pathways involved in the classification of carbohydrate metabolism. **f** Based on the data from omics above, schematic representation of core metabolic and transcriptional relevant differences in the pathway of “fructose and mannose metabolism”. The circles present metabolites, and the rectangles present genes. Red indicates a significant increase in metabolite concentrations or gene expression levels, while blue indicates no detection. **g**–**i** The concentrations of sorbitol (**g**), fructose (**h**) and ATP (**i**) in the hippocampus of db/db and m/m mice (*n* = 6). **j**–**m** Representative western blot (**j**) and densitometric analysis of AR (**k**), GLUT5 (**l**) and KHK (**m**) (*n* = 6). The data are presented as the mean ± SEM and were analyzed by Student’s *t* test (**g**–**i**, **k**–**m**). *P < 0.05; **P < 0.01; ***P < 0.001; ****P < 0.0001.

Periodic measurements of cognitive status demonstrated marked and persistent cognitive dysfunction in db/db mice compared with the m/m mice at 24 weeks (Supplementary Fig. 1). Consequently, widely targeted metabolomic analysis was performed on serum and hippocampus to systematically analyze metabolic changes between db/db mice and m/m mice at that age. A total of 481 metabolites were identified by serum metabolomics analysis, including 190 upregulated and 49 downregulated metabolites (fold change (FC) > 1.2 or < 0.83, variable importance in projection (VIP) > 1 and *P* < 0.05) (Supplementary Fig. 2a). In the hippocampus, 526 metabolites were identified, including 182 upregulated and 89 downregulated metabolites (Supplementary Fig. 2b). Among them, glucose levels were upregulated in both the hippocampus and the serum of db/db mice, while the levels of sorbitol, an intermediate metabolite in the conversion from glucose to fructose, were only elevated in the hippocampus (Fig. 1a), indicating the activation of the polyol pathway, at least in the hippocampus, in the diabetic mouse model.

To investigate the molecular aspects of the metabolic perturbations, we next performed proteomic and RNA-seq analyses in the hippocampus. Compared with m/m mice, proteomic analysis yielded 21 upregulated and 15 downregulated proteins among 5398 proteins from db/db mice (FC > 1.2 or < 0.83 and *P* < 0.05) (Supplementary Fig. 2c). Gene set enrichment analysis (GSEA) revealed that the “fructose and mannose metabolism” pathway was enriched at the top of the ranked list, and the normalized enrichment score (NES) was 1.77 in diabetic mice (Fig. 1b, *P* = 0.0038). Transcriptomic analysis detected 55,450 genes, of which 9,323 were upregulated and 8,878 were downregulated (FC > 1.2 or < 0.83 and *P* < 0.05) (Supplementary Fig. 2d). Subsequent GO analysis revealed that some differentially expressed genes (DEGs) were enriched in pathways related to fructose transport, microglial function, mitochondria and ROS metabolism (Supplementary Fig. 2e). In addition, genes in the “fructose and mannose metabolism” pathway (Supplementary Fig. 3) and *Slc2a5* (protein name: GLUT5), a gene that encodes the major transporter of fructose^28^, were increased in the hippocampus of db/db mice (Fig. 1c). Furthermore, comprehensive analysis of transcriptomics and metabolomics datasets was performed. KEGG enrichment analysis of the metabolomics and transcriptomic data showed that the “carbohydrate metabolism” pathway ranked second among the major metabolic processes (Fig. 1d), while the “fructose and mannose metabolism” pathway ranked third among all carbohydrate metabolism pathways (Fig. 1e), with 4 metabolites and 15 genes significantly altered (Supplementary Fig. 4). In particular, the levels of key metabolites, such as glucose, sorbitol, and genes within metabolic pathways, such as *Slc2a5* and *Khk*, were elevated (Fig. 1f). Taken together, transcriptome, metabolomics and proteomics analyses all indicated that endogenous fructose metabolism was activated in db/db mice.

To further assess whether the fructokinase pathway was activated in the db/db mice, the levels of sorbitol and fructose in the hippocampus were determined. The results demonstrated that the sorbitol and fructose levels were elevated in the db/db mice. However, the level of ATP, a unique characteristic of fructose metabolism^8^, was reduced (Fig. 1g–i), which is consistent with studies in liver and renal models^10,29^. In addition, the expression of AR, KHK and GLUT5 was also significantly increased in the hippocampus of db/db mice (Fig. 1j–m), indicating the activation of the fructokinase pathway.

### Cerebral endogenous fructose is predominantly metabolized in microglia by KHK

**Fig. 2:**
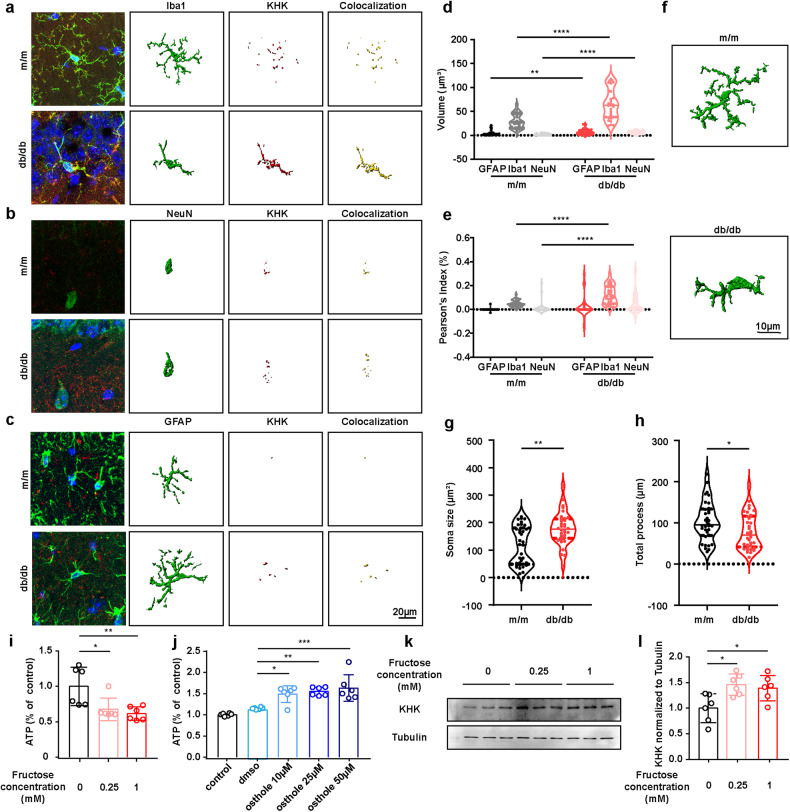
The central role of microglia in fructose metabolism in the hippocampus of db/db mice. **a**–**c** Representative confocal images and three-dimensional reconstruction by Imaris software illustrated the colocalization status of KHK (red) in microglia (**a**, green), neurons (**b**, green) or astrocytes (**c**, green). DNA was labeled by DAPI (blue). Scale bar = 20 μm. **d** Volume of KHK and cell marker colocalization intensity from (**a**–**c**) (*n* = 30 from 4-5 mice). **e** Correlation of KHK with different cell markers based on fluorescence intensity (*n* = 30 from 4-5 mice). **f** Representative Imaris-based three-dimensional reconstruction of hippocampal microglia from db/db and m/m mice. Scale bar = 10 μm. **g**, **h** Quantification of microglial soma size (**g**) and total processes (**h**) (*n* = 34 microglia from 4-5 mice). **i** The alteration of ATP levels in microglia treated with increasing fructose doses (*n* = 6). **j** Osthole increased ATP levels in microglia treated with 0.25 mM fructose (*n* = 6). **k**, **l** Representative western blot (**k**) and densitometric analysis (**l**) of KHK (*n* = 6). The data are presented as the mean ± SEM. The data in (**d**, **e**) were analyzed by Student’s *t* test or the Mann‒Whitney test based on the results of the normal distribution test. The data in (**g**) were analyzed by the Mann‒Whitney test. The data in (**h**) were analyzed by Student’s *t* test. The data in (**i**, **j**, **l**) were analyzed by one-way ANOVA with Tukey’s post hoc analysis. *P < 0.05; **P < 0.01; ***P < 0.001; ****P < 0.0001.

To identify the specific expression of KHK and GLUT5 in the hippocampus, single-cell RNA-Seq (sc RNA-Seq) data (GSE201644) between db/db and db/m mice in the GEO database (http://www.ncbi.nlm.nih.gov/geo) were acquired and analyzed^25^. The cells were divided into 13 distinct cell types based on cell-type-specific gene expression and annotated as shown in Supplementary Fig. 5a, b. Both *Khk* and *Slc2a5* (GLUT5) were expressed mainly in microglia (Supplementary Fig. 6a, b, d, e), and their expression increased in db/db mice compared with db/m mice (Supplementary Fig. 6c, f). Consistent with previous reports that GLUT5 is primarily expressed in microglia^22^, we found strong GLUT5 (Supplementary Fig. 7) and KHK (Fig. 2a–e) immunostaining signals that were predominantly colocalized with Iba-1 rather than GFAP or NeuN in the hippocampus of db/db mice, suggesting that microglia are the major site of endogenous fructose metabolism in the brain. Moreover, quantitative morphometric analysis showed that the microglia of db/db mice featured increased soma size and reduced branch complexity, implying the involvement of microglial activation in DACD pathogenesis (Fig. 2f–h). In the BV2 cell line, ATP levels declined in a fructose dose-dependent manner (Fig. 2i) and could be blocked by the KHK inhibitor osthole (Fig. 2j). In addition, increasing fructose doses induced the expression of KHK (Fig. 2k, l). Altogether, these results suggest that microglia play a key role in endogenous fructose metabolism.

### *Khk* knockdown blocks endogenous fructose metabolism

To assess the effect of endogenous fructose metabolism in microglia, we sought to suppress KHK expression in the hippocampus using the recombinant adeno-associated virus rAAV-U6-shRNA-*Khk*-CMV-EGFP-SV40pA (AAV-shRNA-*Khk*). The infection efficiency of AAV in the hippocampus was confirmed by the expression of EGFP, and KHK expression was efficiently knocked down by shRNA (Supplementary Fig. 8a–c). As a consequence, *Khk* knockdown had no apparent effect on body weight, water intake, serum glucose, serum fructose, serum sorbitol, fasting glucose, fasting insulin, or homeostatic model assessment of insulin resistance (HOMA-IR) (Supplementary Fig. 8d–k). However, *Khk* knockdown resulted in a substantial increase in ATP levels, further indicating the inhibition of endogenous fructose metabolism in the hippocampus (Supplementary Fig. 8l).

### *Khk* knockdown reduces microglial NOX4 expression both in vivo and in vitro

**Fig. 3:**
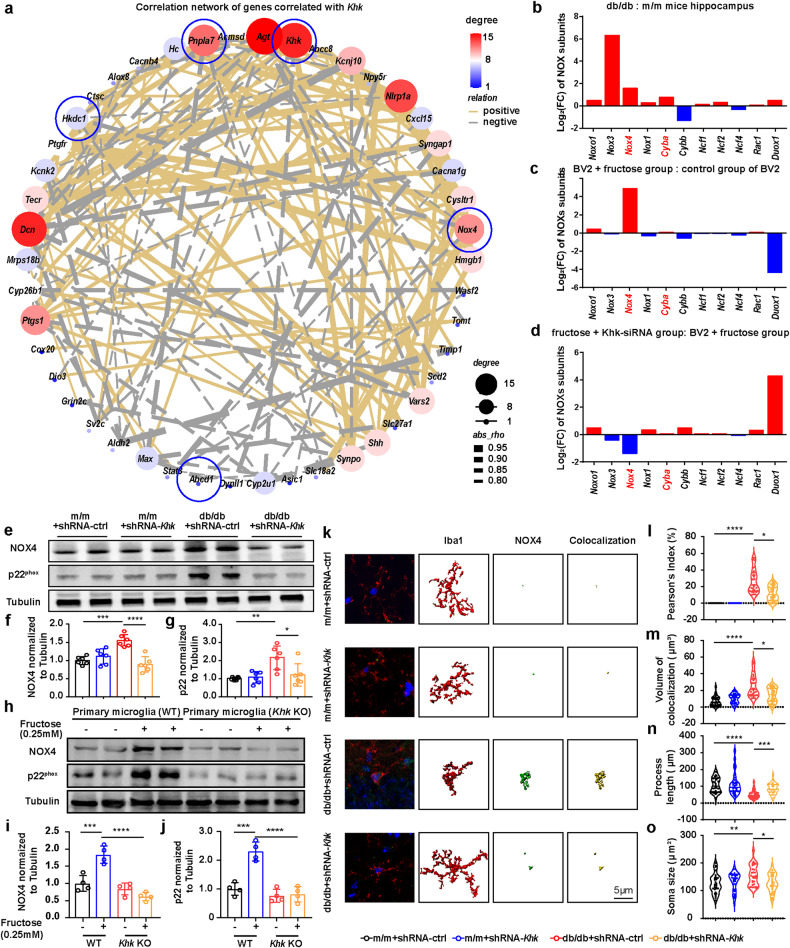
Effects of *Khk* knockdown on NOX4 expression and ROS production. **a** Correlation networks of genes correlated with *Khk* in hippocampal RNA-seq analysis based on the |correlation coefficient |> 0.8. **b** Fold changes in *Nox* subunits from RNA-seq analysis of the hippocampus (db/db vs. m/m mice) (*n* = 5). **c**, **d** Fold changes in *Nox* subunits from RNA-seq analysis in the BV2 cell line between different conditions (*n* = 6). **e**–**g** Representative western blot (**e**) and densitometric analysis of NOX4 (**f**) and p22^phox^ (**g**) in the hippocampus (*n* = 6). **h**–**j** Representative western blot (**h**) and densitometric analysis of NOX4 (**i**) and p22^phox^ (**j**) in WT or *Khk* KO primary microglia with or without fructose treatment (*n* = 4). **k** Representative confocal images and three-dimensional reconstruction of NOX4 (green) in microglia (red) (*n* = 30). DNA was labeled by DAPI (blue). Scale bar = 5 μm. **l** Correlation of NOX4 and Iba1 fluorescence intensity (*n* = 30 from 4-5 mice). **m** Volume of NOX4 and Iba1 colocalization (*n* = 30 from 4-5 mice). **n**, **o** Quantification of microglial total processes (**n**) and soma size (**o**) (*n* = 30 from 4-5 mice). The data are presented as the mean ± SEM and were analyzed by one-way ANOVA with Tukey post hoc analysis (**f**, **g**, **i**, **j**) or by Kruskal‒Wallis followed by Dunn’s multiple comparisons tests (**l**–**o**). *P < 0.05; **P < 0.01; ***P < 0.001; ****P < 0.0001.

Next, we sought to explore the downstream effectors altered by endogenous fructose metabolism. To this end, we first performed a correlation analysis on hippocampal transcriptomic data. The results demonstrated that 287 DEGs were correlated with *Khk*, 24 of which were negatively correlated with *Khk* and enriched in the pathway related to synapse and mitochondrion, while another 21 DEGs were positively correlated with *Khk* and enriched in the pathway of “aging”, “inflammatory response” and “response to axon injury” (Supplementary Fig. 9). In addition, based on the |correlation coefficient |> 0.8, correlation networks indicated that *Khk* was positively correlated with *Nox4* (Fig. 3a). NOX4 requires p22^phox^ to form an active NADPH oxidase complex^14^. Indeed, most NOX enzyme genes were upregulated in the hippocampus of db/db mice (Fig. 3b), with *Nox4* and *Cyba* (protein name: p22^phox^) ranking second and third, respectively. Furthermore, we performed RNA-seq analysis of the BV2 cell line treated with or without fructose and *Khk* depletion (FC > 1.2 or < 0.83 and *P* value < 0.05, Supplementary Fig. 10a, b). Similarly, fructose loading increased *Nox4* expression, while silencing *Khk* reduced it, although the trend of *Cyba* was not very clear (Fig. 3c, d). Western blot analysis also confirmed that NOX4 and p22^phox^ were upregulated in the hippocampus of db/db mice and microglia (wild-type (WT) primary microglia and BV2 cells) treated with fructose but were recovered in *Khk* knockdown mice, *Khk* KO microglia and BV2 cells treated with *Khk*-siRNA (Fig. 3e–j, Supplementary Fig. 11a–c). In concordance with these reports, three-dimensional reconstruction immunofluorescence staining showed that NOX4 levels were enhanced in the microglia of the db/db mice but significantly decreased with *Khk* depletion (Fig. 3k–m). Simultaneously, db/db mice developed abnormal microglia characterized by reduced branch complexity and increased soma size, while loss of KHK partially restored microglial morphology (Fig. 3n, o). Taken together, these results indicated that KHK depletion led to inhibition of NOX4 signaling in microglia.

### *Khk* knockdown inhibits NOX4 mitochondrial translocation and recovers mitochondrial homeostasis in microglia

**Fig. 4:**
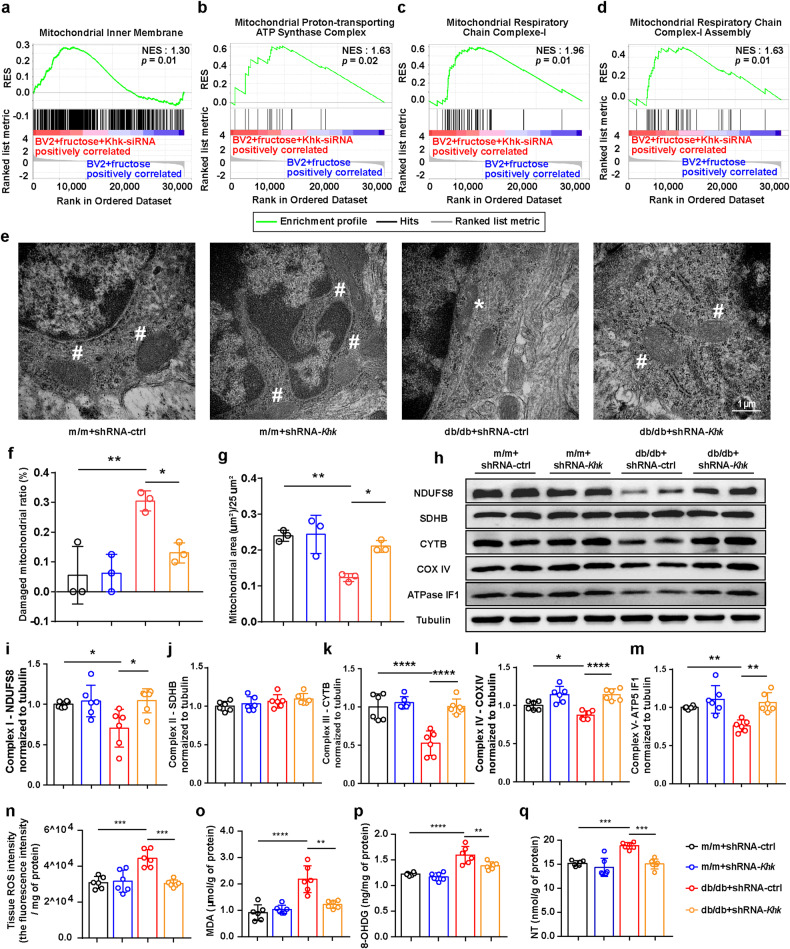
*Khk* knockdown alleviated mitochondrial injury in microglia and resisted oxidative damage. **a**–**d** The alterations of pathways in the RNA-seq of BV2+fructose+*Khk*-siRNA vs. BV2+fructose group via GSEA (*n* = 6). RES = Running Enrichment Score. **e** Transmission electron microscopy showing the mitochondrial morphology of microglia in the hippocampus (*n* = 3). White asterisks represent damaged mitochondria, while pounds represent healthy mitochondria. Scale bar = 1 μm. **f**, **g** Quantification of the damaged mitochondria ratio (**f**) and mitochondrial area (g) in hippocampal microglia. **h**–**m** Representative western blot (**h**) and densitometric analysis of OXPHOS-related proteins, including NDUFS8 (complex I, **i**), SDHB (complex II, **j**), CYTB (complex III, **k**), COX IV (complex IV, **l**) and ATPase IF1 (complex V, **m**), in the hippocampus (*n* = 6). **n**–**q** Effects of KHK knockdown on redox homeostasis, such as ROS (**n**), MDA (**o**), 8-OHDG (**p**), and NT (**q**), in the hippocampus of m/m or db/db mice (*n* = 6). The data in (**f**, **g**, **i**–**q**) are presented as the mean ± SEM and were analyzed by one-way ANOVA with Tukey’s post hoc analysis. *P < 0.05; **P < 0.01; ***P < 0.001; ****P < 0.0001.

In the correlation networks of DEGs between db/db mice and m/m mice, *Nox4* was not only positively correlated with *Khk* but also negatively correlated with genes related to mitochondria, including *Pnpla7*, *Hkdc1* and *Abcd1* (Fig. 3a and Supplementary Fig. 9). Given that GSEA revealed that silencing *Khk* upregulated the genes that facilitate mitochondrial function in BV2 cells (Fig. 4a–d. Supplementary Fig. 10c, d), we sought to detect whether enhanced endogenous fructose metabolism damaged mitochondria in microglia. Transmission electron microscopy results showed that the ratio of damaged mitochondria in microglia was increased in db/db mice and restored when fructose metabolism was blocked through *Khk* depletion (Fig. 4e–g). Next, we detected the protein levels of oxidative phosphorylation complex members and found that the expression of NDUFS8 (Complex I), CYTB (Complex III), COX IV (Complex IV) and ATPase IF1 (Complex V), but not SDHB (Complex II), was significantly decreased in the hippocampus of db/db mice, while deleting *Khk* restored these protein levels (Fig. 4h–m). Additionally, all of the oxidative phosphorylation complex members were decreased in WT primary microglia treated with fructose and recovered in primary microglia from *Khk* KO mice (Supplementary Fig. 12a–f). In addition, the levels of ROS, NT, MDA and 8-OHDG, which represent ROS generation and release, peroxynitrite-mediated tyrosine nitration, intracellular lipid oxidation and DNA oxidative lesions, were also elevated in the hippocampus of db/db mice and restored by knockdown of *Khk* (Fig. 4n–q), indicating that endogenous fructose metabolism is sufficient for microglial mitochondrial damage in db/db mice.

**Fig. 5:**
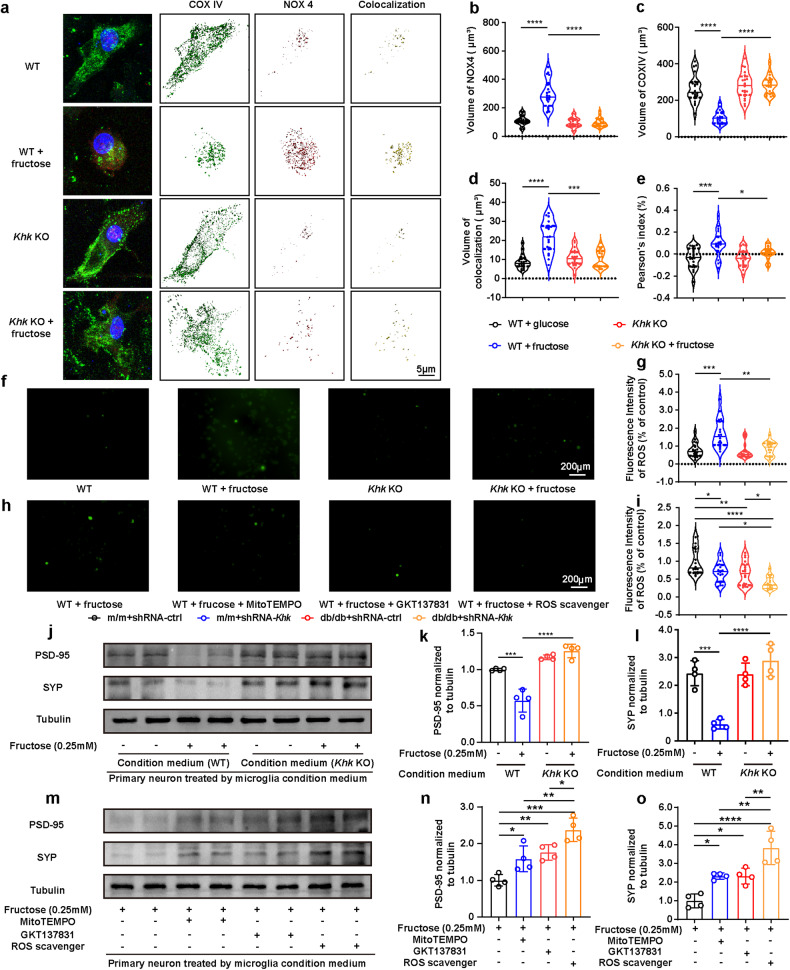
Fructose metabolism in microglia mediates spine elimination via NOX4 mitochondrial translocation and ROS generation. **a** Representative immunofluorescence image and three-dimensional reconstruction of the mitochondrial markers COX IV (green) and NOX4 (red) in WT or *Khk* KO primary microglia treated with or without fructose. DNA was labeled by DAPI (blue). Bar = 5 μm. **b**–**d** Fluorescence intensity of NOX4 (**b**), COX IV (**c**) and their colocalization (**d**) (*n* = 20 cells from 3 independent experiments). **e** Correlation of NOX4 and COX IV fluorescence intensity (*n* = 20 cells from 3 independent experiments). **f**, **g** Representative fluorescence images (**f**) and quantification analysis (**g**) of ROS fluorescence intensity in primary microglia treated with or without fructose (*n* = 18 from 3 independent experiments). Scale bar = 200 μm. **h**, **i** Representative fluorescence images (**h**) and quantification analysis (**i**) of ROS fluorescence intensity of primary microglia treated with or without NOX4 inhibitor (GKT137831), mitochondrial ROS inhibitor (Mito-Tempo) or ROS scavenger (*N*-acetylcysteine) in fructose-loaded medium (*n* = 18 from 3 independent experiments). Scale bar = 200 μm. **j**–**l** WT or *Khk* KO primary microglia were treated with or without fructose, and then the primary microglia-conditioned medium was collected for primary neuron culture. Representative western blot (**j**) and densitometric analysis of PSD-95 (**k**) and SYP (**l**) in primary neurons (*n* = 4). **m**–**o** WT primary microglia were treated with or without MitoTEMPO, GKT137831, and ROS scavengers in fructose-loaded medium, and the primary microglia-conditioned medium was collected for primary neuron culture. Representative western blot (**m**) and densitometric analysis of PSD-95 (**n**) and SYP (**o**) expression in primary neurons (*n* = 4). The data are presented as the mean ± SEM and were analyzed by one-way ANOVA with Tukey post hoc analysis (**c**, **e**, **g**, **k**, **l**, **n**, **o**) or Kruskal‒Wallis followed by Dunn’s multiple comparisons tests (**b**, **d**, **i**). *P < 0.05; **P < 0.01; ***P < 0.001; ****P < 0.0001.

Notably, in fructose-treated primary microglia and BV2 cells, three-dimensional reconstruction images demonstrated that *Khk* knockdown or knockout elevated the intensity of COX IV but reduced the intensity of NOX4 and the colocalization of NOX4 and COX IV (Fig. 5a–e, Supplementary Fig. 13a–e). Additionally, treatment with fructose elevated ROS production in primary microglia and BV2 cells, while knocking out *Khk*, silencing *Khk* or applying a NOX4 inhibitor (GKT137831), mitochondrial ROS inhibitor (Mito-Tempo) and ROS scavenger (N-acetylcysteine) reduced ROS production (Fig. 5f–i, Supplementary Fig. 13f–i). Collectively, endogenous fructose metabolism promoted NOX4 mitochondrial translocation and destroyed mitochondrial homeostasis in microglia, which could be reversed by *Khk* depletion.

### *Khk* depletion restores structural synaptic plasticity by reducing microglial ROS production

**Fig. 6:**
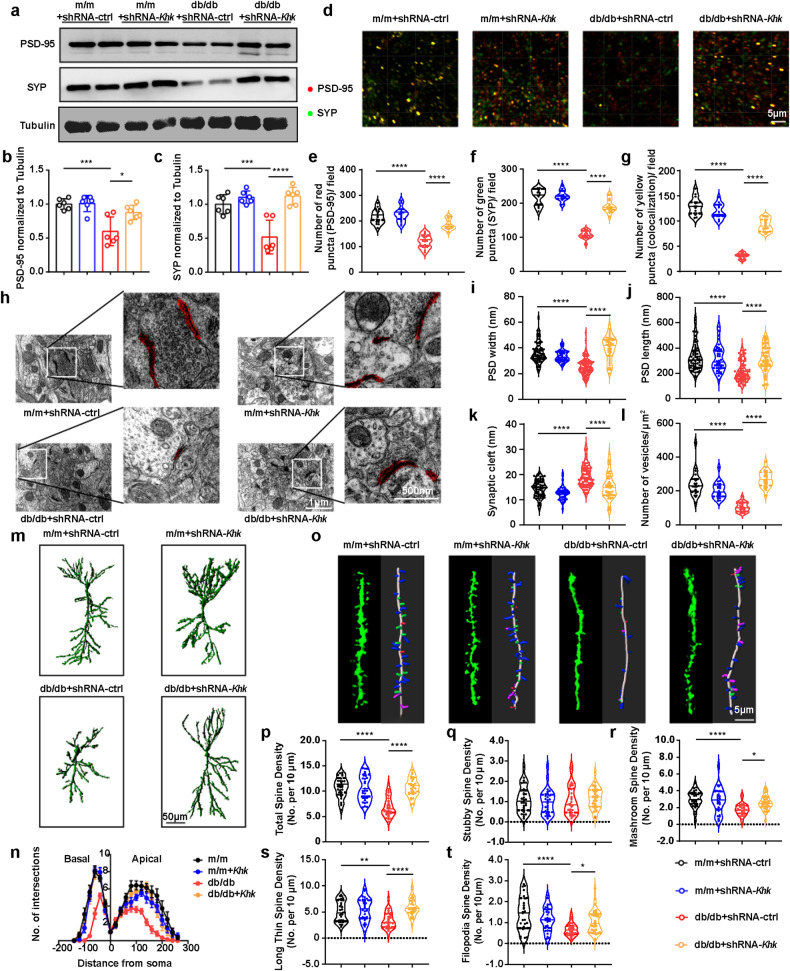
Effects of *Khk* knockdown on structural synaptic plasticity in the hippocampus of db/db mice. **a**–**c** Representative western blot (**a**) and densitometric analysis of PSD-95 (**b**) and SYP (**c**) (*n* = 6). **d**–**g** Immunofluorescence (**d**) and quantitative analysis of PSD-95-positive (red) (**e**), SYP-positive (green) (**f**) or double-positive puncta (yellow) (g) (*n* = 15 from 3–5 mice). Scale bar = 5 μm. **h**–**l** Representative images of synaptic ultrastructure (**h**, highlighted in red) and quantitative analysis of PSD width (**i**) (*n* = 44–82 from 3-5 mice) and length (**j**) (*n* = 44–82 from 3-5 mice), synaptic cleft (**k**) (*n* = 44-82 from 3-5 mice) and number of vesicles (**l**) (*n* = 20–23 from 3-5 mice). Scale bar = 500 nm. **m**, **n** Golgi staining imaging of neuronal morphology (**m**) and quantification of dendritic complexity (**n**) (*n* = 15 from 3-5 mice). Scale bar = 50 μm. **o** Representative three-dimensional reconstruction images of dendrites (left) and different kinds of spines in hippocampal pyramidal neurons (right) (*n* = 30 from 3-5 mice). Scale bar = 5 μm. **p**–**t** Quantification of different kinds of spine densities, including total spine density (**p**) and stubby (**q**), mushroom (**r**), long thin (**s**) and filopodia spines (**t**) (*n* = 30 from 3-5 mice). The data are presented as the mean ± SEM and were analyzed by one-way ANOVA with Tukey post hoc analysis (**b**–**g**, **r**, **t**) or Kruskal‒Wallis followed by Dunn’s multiple comparisons tests (**i**–**l**, **p**, **q**, **s**). The data in (**n**) were analyzed by two-way ANOVA. *P < 0.05; **P < 0.01; ***P < 0.001; ****P < 0.0001.

Our RNA-seq results showed that the abundance of synapse-related genes had a decreasing trend in the db/db mice compared with the m/m mice (Supplementary Fig. 14). In addition, *Khk* was negatively correlated with genes enriched in the “synapse” pathway (Supplementary Fig. 9). Considering that microglia-derived ROS are a critical cause of spine elimination^30,31^, we investigated whether endogenous fructose metabolism in microglia damaged synaptic plasticity by ROS production. Conditioned medium from wild-type primary microglia treated with fructose decreased the expression of synaptic proteins in primary neurons, while medium from *Khk* KO primary microglia or medium from wild-type primary microglia treated with the NOX4 inhibitor GKT137831, the mitochondrial ROS inhibitor Mito-Tempo or the ROS scavenger N-acetylcysteine restored synaptic protein levels (Fig. 5j–o), indicating a causal role of fructose metabolism in damaging synaptic plasticity via microglia-derived ROS. Similar results were also observed in the HT22 cell line treated with BV2 conditioned medium (Supplementary Fig. 15). In the hippocampus of db/db mice, western blotting showed that *Khk* knockdown reversed the reduction in postsynaptic density protein 95 (PSD-95) and synaptophysin (SYP) (Fig. 6a–c). Consistently, immunofluorescence analysis revealed an increased number of PSD-95^+^, SYP^+^ or PSD-95^+^/SYP^+^ double-positive puncta in the CA1 area upon *Khk* knockdown in db/db mice (Fig. 6d–g). Furthermore, transmission electron microscopy showed that the db/db mice had reduced PSD length and width, increased synaptic cleft and a reduced number of presynaptic vesicles per synapse in comparison with the m/m mice, which could be reversed by *Khk* knockdown (Fig. 6h–l). Moreover, Golgi staining and three-dimensional reconstruction demonstrated that the dendritic complexity, total spine density and the density of stubby, long thin, mushroom and filopodia were decreased in db/db mice, while these defects were recovered by *Khk* depletion (Fig. 6m–t). Of interest, although the “scavenger receptor activity” pathway was enriched by DEGs in RNA-seq (Supplementary Fig. 2e), PSD-95 puncta internalization in microglia was not altered by knocking down *Khk*, indicating that the elimination of spines may not be related to microglial engulfment (Supplementary Fig. 16). Overall, inhibition of endogenous fructose metabolism could mitigate structural synaptic plasticity deficits of CA1 pyramidal neurons via reduction of microglia-derived ROS in db/db mice.

### Loss of KHK rescues aberrant electrophysiology of CA1 pyramidal neurons and cognitive dysfunction in db/db mice

**Fig. 7:**
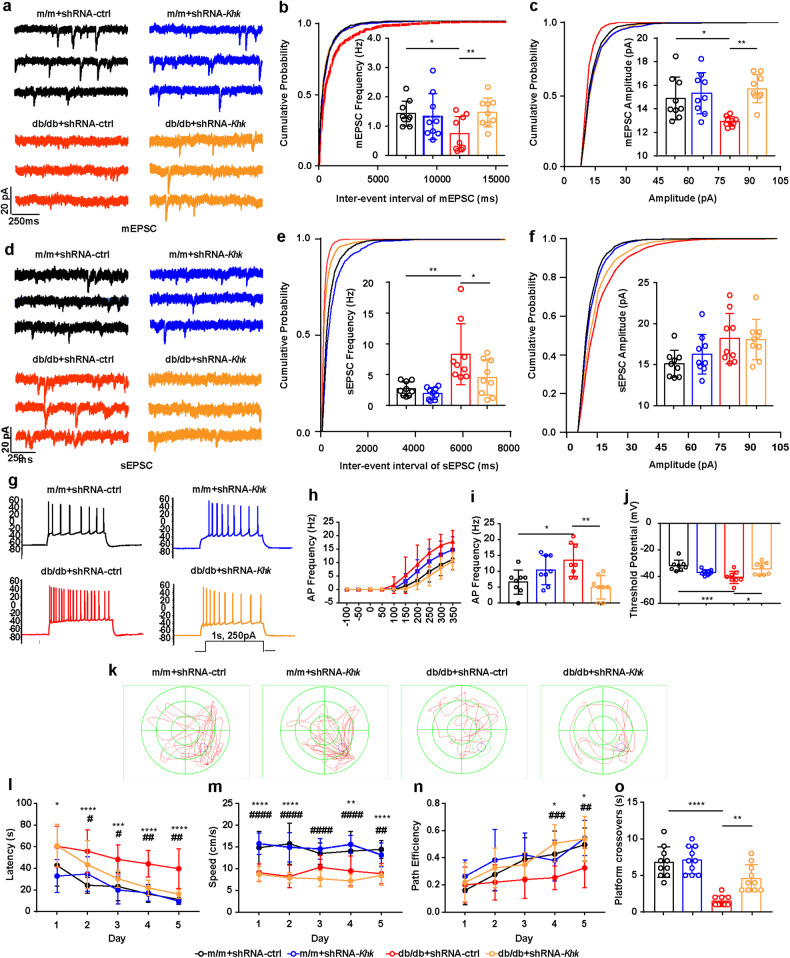
Effects of *Khk* knockdown on synaptic transmission. **a**–**c** Representative trace of mEPSCs (**a**) and quantification analysis of frequency (**b**) and amplitude (**c**) alterations in mEPSCs. (*n* = 9). **d**–**f** Representative trace of sEPSCs (**d**) and quantification analysis for frequency (**e**) and amplitude (**f**) alternations of sEPSCs (*n* = 9). **g**, **h** Representative trace of action potentials (APs) (**g**) and their frequency (**h**) in different groups. **i**, **j** Quantitative analysis of AP frequency (**i**) and threshold potential (**j**) (*n* = 8). **k**–**o** Representative traces from the Morris water maze (MWM) test (**k**) and the quantification of latency (**l**), swimming speed (**m**), path efficiency (**n**), and platform crossovers (**o**) (*n* = 10). The data are presented as the mean ± SEM. The data in (**b**, **c**, **e**, **f**, **i**, **j**, **o**) were analyzed by one-way ANOVA with Tukey’s post hoc analysis. The data in (**l**–**n**) were analyzed by two-way ANOVA. *P < 0.05 versus the m/m + shRNA-ctrl group, ^#^*P* < 0.05 versus the db/db + shRNA-ctrl group; **P or ^##^*P* < 0.01; ***P or ^###^*P* < 0.001; ****P or ^####^*P* < 0.0001.

Spine morphology reflects synaptic function and is necessary for learning and memory formation^32^. Our previous studies and several other studies have reported learning and memory deficits in a diabetes mouse model and human patients^2,23,33^. To investigate whether inhibition of KHK could improve excitatory synaptic transmission in db/db mice, we performed whole-cell voltage clamping in the CA1 pyramidal neurons. The frequency of miniature excitatory postsynaptic currents (mEPSCs) was reduced as the cumulative probability curve of the mEPSC interevent interval shifted right in db/db mice, while the alteration was recovered by *Khk* depletion (Fig. 7a, b). Simultaneously, in db/db mice, the mEPSC amplitude decreased, and the cumulative probability distribution of the mEPSC amplitude shifted to the left; both were restored by Khk knockdown (Fig. 7c). These observations suggested that loss of KHK effectively ameliorated the deficits in synaptic transmission in db/db mice, which was consistent with the recovery of synaptic structure observed before (Fig. 6h–t). Although there was no difference in the amplitude of spontaneous excitatory postsynaptic currents (sEPSCs) between db/db and m/m mice, the frequency of sEPSCs increased as the cumulative probability curve of the sEPSC interevent interval shifted to the left, which was alleviated after *Khk* depletion (Fig. 7d–f). Due to increased neuronal excitability in db/db mice^34^, we speculated that the elevation of sEPSCs may be associated with increased AP frequency. Our results demonstrated that the AP frequency increased and the threshold potential level slightly declined in the db/db mice, whereas the parameters recovered after *Khk* depletion (Fig. 7g–j).

To investigate whether the electrophysiological changes were related to cognitive ability, the Morris water maze test was used and demonstrated that *Khk* depletion significantly improved cognitive function. *Khk* knockdown decreased escape latency in the 5-day navigation test (Fig. 7l) and elevated platform crossovers on the probe trial day (Fig. 7k, o). To avoid the biases in which the lower swimming velocity of db/db mice might prolong latency (Fig. 7m), the path efficiency, calculated by the distance between the entry and endpoints over the total distance^23^, was also applied to evaluate cognition of the db/db mice. The db/db mice had significantly lower path efficiency from the fourth day of the Morris water-maze test compared with that in the m/m group, which was improved by the depletion of KHK (Fig. 7n). Overall, the loss of KHK expression rescued spine deficits, abnormal electrophysiology and cognitive dysfunction in db/db mice.

## Discussion

**Fig. 8:**
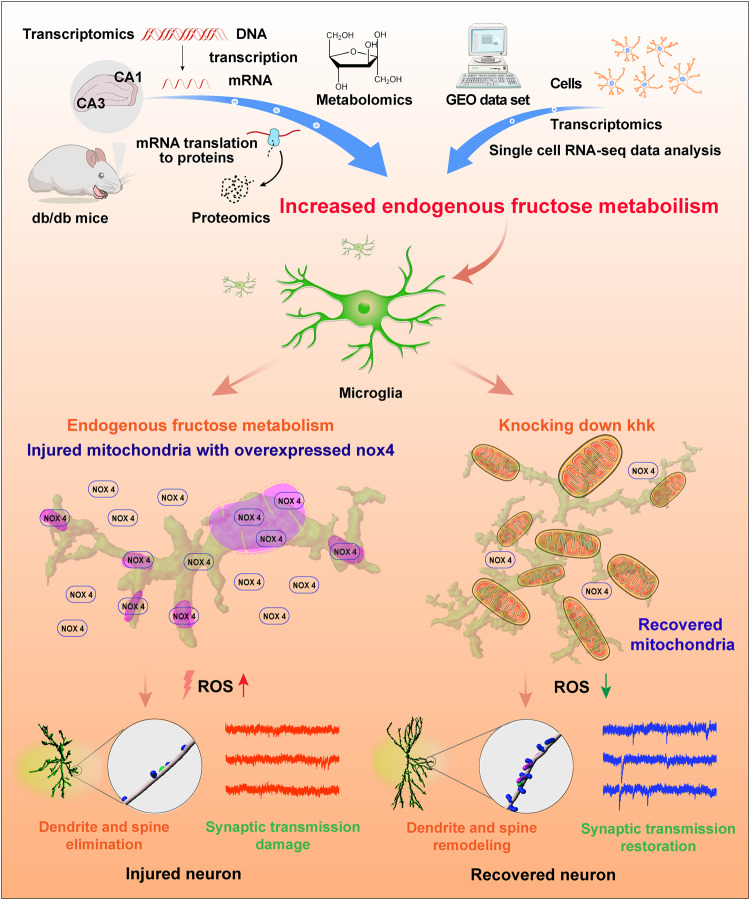
Graphic summary illustration. Multiomics analysis of the mouse hippocampus demonstrated that endogenous fructose metabolism was increased in T2DM animals. Further experiments illustrate that endogenous fructose metabolism occurs predominantly in microglia of the hippocampus and is boosted during T2 diabetes mellitus, leading to the overexpression and mitochondrial translocation of NOX4. Translocated NOX4 injures mitochondria and consequently destroys microglial redox hemostasis. Overproduction of ROS from microglia mediates synaptic deficits and promotes the development of DACD. Knocking down *Khk* downregulates NOX4 expression and mitochondrial translocation and reduces ROS generation in microglia, which in turn restores synaptic impairment and alleviates DACD.

DACD is becoming a public health concern due to its high prevalence and serious consequences, such as worse diabetes management, more frequent occurrence of severe hypoglycemic episodes, and even increased risk of cardiovascular events and death^35,36^. A previous study reported that in China, serum fructose levels are a risk factor for T2 diabetes mellitus and are associated with diabetic nephropathy, retinopathy, and neuropathy^6^. Although the enhanced metabolism of dietary fructose is strongly associated with cognitive deficits^37^ and the polyol pathway seems neurotoxic because its activation is negatively correlated with cognition^21^, the crosstalk between endogenous fructose metabolism and its role in pathogenesis in the brain are still less defined. Here, we report that cerebral endogenous fructose is predominantly metabolized in microglia by KHK, which exacerbates microglial activation and synaptic damage in DACD. Depleting *Khk* reduced NOX4 expression and its mitochondrial translocation, resulting in a decline in ROS generation in microglia, which in turn restored synaptic structural plasticity impairment and aberrant electrophysiology and alleviated diabetes-associated cognitive dysfunction-like phenotypes in db/db mice (Fig. 8).

Fructose is distinct from all other nutrients, as its metabolism triggers the organism to go into a ‘safety mode’ to store energy^5^. However, with the improvement of material conditions, excessive caloric intake has led to glucose supply-demand mismatch, and a saturated glycolytic pathway promotes excess glucose to be metabolized via the polyol pathway. Thus, excessive fructose metabolism shifts the body from “safety mode” to “disease initiation” and is associated with metabolic syndrome, NAFLD, T2 diabetes mellitus, kidney disease and cardiovascular disease^5^. Given that the production of fructose in the central nervous system has been reported in diabetic organisms of several species^17,38–40^ and is negatively correlated with cognitive function^21^, the hypothesis that cerebral fructose metabolism could be a pivotal initiating pathway for cognitive impairment has previously been proposed^8,41^. Our multiomics analysis, including analysis of the hippocampus metabolome, transcriptome, and proteome, demonstrated that the “fructose and mannose metabolism” pathway is activated in the hippocampus of db/db mice. Increased expression of AR, GLUT5 and KHK, elevated hippocampal sorbitol and fructose levels, and decreased hippocampal ATP levels in the db/db mice further confirmed activation of the fructokinase pathway. Collectively, we comprehensively demonstrated that endogenous fructose metabolism is enhanced in the hippocampus of db/db mice.

Fructose can be metabolized by KHK^42^, the cellular localization of which in the hippocampus is unclear. GLUT5 is the fructose transporter expressed in human and rat microglia, and its function in such cells remains unknown^43^. Given that high levels of KHK and GLUT5 were identified as molecular signatures of active fructose metabolism^44^, combined with our results that both KHK and GLUT5 were upregulated in db/db mice and that single-cell RNA-seq analysis from the GEO dataset revealed that *Khk* and *Slc2a5* were mainly expressed in microglia and elevated in the hippocampus of db/db mice, we speculated that microglia might be the major participants in endogenous fructose metabolism in the hippocampus. Three-dimensional reconstruction of colocalization between KHK, GLUT5 and markers of neurons (NeuN), astrocytes (GFAP), or microglia (Iba1) in the hippocampus demonstrated that both KHK and GLUT5 were predominantly located in microglia and were upregulated in db/db mice. Simultaneously, the activation of microglia in the hippocampus of db/db mice was evidenced by the amoeboid shape with reduced processes. In vitro, the expression of KHK in BV2 cells was upregulated by fructose loading and showed a ceiling effect at 0.25 mM, which is very close to the physiological levels of circulating fructose of 0.04 to 0.2 mM^45^. In addition, fructose load caused decreased ATP levels, which could be restored by osthole, an inhibitor of KHK^29^. Collectively, these results indicate that fructose is mainly metabolized in microglia, which are activated in the hippocampus of db/db mice.

Because inhibition of fructose metabolism prevents hepatic metabolic syndrome and restores chronic renal injury in diabetes or acute kidney injury^10,11,46^, *Khk* was depleted in the bilateral hippocampus of db/db mice. The increased ATP level in the hippocampus indicated the blockage of fructose metabolism. Notably, although *Khk* knockout mice were protected against an increase in energy intake, body weight, and elevated blood insulin and glucose levels after exposure to 10% glucose for 14 weeks^11^, *Khk* knockdown in the hippocampus did not improve these metabolic parameters.

In Alzheimer’s disease and other neurodegenerative disorders, microglia are activated and release excessive ROS^47^ because they highly express superoxide-producing NOXs^48^, which produce O^2–^ or H_2_O_2_, the major sources of ROS^49^. In line with the evidence that fructose metabolism generates 100 times more ROS than glucose^50^ and can enhance the expression of ROS-producing NOXs in human monocytes and mouse macrophages^45^, our RNA-seq analysis of the hippocampus revealed that db/db mice expressed higher levels of NOXs than m/m mice and that *Nox4* expression was positively associated with *Khk* expression. Further RNA-seq analysis of the BV2 cell line also showed that fructose loading increased *Nox4* expression, while silencing *Khk* reduced Nox4 expression. The activity of NOX4 is primarily regulated by its expression level^49^ and requires the catalytic subunit p22^phox^ ^51^. Subsequent western blot experiments confirmed that the expression of NOX4 and p22^phox^ increased, while silencing and knocking out *Khk* reduced it both in vivo and in vitro. Immunofluorescence and three-dimensional reconstruction also showed that *Khk* depletion reduced the colocalization of NOX4 with Iba1 and turned amoeboid-shaped microglia into those with a small soma and longer processes. The potential mechanism by which endogenous fructose metabolism upregulates NOX4 may be through NF-κB-mediated transcription^49^, as fructose metabolism can enhance the nuclear expression of NF-κB p65 in the hippocampus^52^, and our GO enrichment in RNA-seq of the hippocampus also showed that the “positive regulation of NF-kappaB transcription factor activity” pathway was enriched in db/db mice compared to m/m mice (Supplementary Fig. 2e). Above all, these observations suggested that endogenous fructose metabolism led to the upregulation of NOX4 in microglia.

Our previous studies have confirmed that mitochondrial damage is the key pathogenic factor of DACD, and restoring mitochondrial homeostasis, especially redox homeostasis, is the key to ameliorating DACD^23,33^. The expression of oxidative phosphorylation-related proteins was dramatically reduced in db/db mice but was restored by *Khk* knockdown, which was associated with a decline in ROS, 8-OHDG, MDA, and NT. Additionally, knocking out *Khk* in primary microglia reversed the reduction in oxidative phosphorylation-related proteins after fructose treatment. Because NOX4 is inversely correlated with the mitochondria-related genes *Pnpla7*, *Hkdc1* and *Abcd1* in the correlation analysis of hippocampal RNA-seq and GSEA of BV2 cell RNA-seq showed that silencing *Khk* downregulated the pathway related to mitochondria, we asked whether and how NOX4 impacted mitochondrial homeostasis.

NOX4 is a major source of ROS in diabetic animals^53^ and has been found to be located in mitochondria in the kidney cortex in diabetes^54^. In adipocytes and hepatocytes, uric acid, one of the main metabolites of fructose, promoted the translocation of NOX4 to mitochondria and impacted redox homeostasis^55,56^. Hence, mitochondrial and NOX-mediated ROS production in activated microglia may be functionally linked^48^, creating a vicious circle. We validated that in microglia, fructose loading elevated the localization of NOX4 on mitochondria, while silencing or knocking out *Khk* reversed this effect. In addition, under fructose loading, both inhibition of NOX4 and mitochondrial ROS production efficiently reduce microglial ROS generation. Collectively, these studies provide the novel finding that endogenous fructose metabolism-boosted microglial ROS generation is, at least partially, associated with mitochondrial NOX4 translocation.

In light of the fact that microglia-derived ROS are the main cause of synaptic deficits^30,31^ and that *Khk* depletion does not affect microglial phagocytosis of psd-95, we further investigated whether endogenous fructose metabolism damages neurons through the generation of ROS. In primary neurons treated with primary microglia-conditioned medium or HT22 cells treated with BV2-conditioned medium, inhibition of both NOX4 and mitochondrial ROS production in microglia improved synaptic protein expression. These results mirror the data from db/db mice showing that depletion of *Khk* reduced ROS levels in the hippocampus accompanied by restoration of synaptic plasticity deficits. Simultaneously, the complexity of dendrites and the densities of mushroom, long thin dendrites and filopodia were increased. Since hippocampal spines are primarily sites of excitatory glutamatergic synaptic transmission^57^, mEPSCs and sEPSCs were recorded, which provided information on action potential-independent (vesicular release) and -dependent (network activity) excitatory synaptic transmission^27^. The decline in the amplitude and frequency of mEPSCs was in accordance with pre- and postsynaptic deficits, which corresponded with the loss of mushroom spines (the mature “memory spine”) in the hippocampus of db/db mice. In contrast, the frequency of sEPSCs was increased, which may be due to the hyperexcitability of neurons in db/db mice^34^. Increased action potential (AP) frequency and decreased threshold potential of CA1 pyramidal neurons validated our assumption. Indeed, both decreased synaptic activity and increased neuronal excitability were associated with impaired learning and memory^58,59^. Although the underlying mechanism may be different, this paradox has also been observed in AD^60^. Fortunately, knocking down *Khk* rescued the electrophysiological abnormalities, further alleviating cognitive dysfunction in db/db mice.

In summary, our study highlights that endogenous fructose metabolism occurs mainly in microglia, leading to the upregulation and mitochondrial translocation of NOX4. *Khk* depletion blocks these effects, reduces excess ROS accumulation and alleviates synaptic damage, finally resulting in the alleviation of DACD.

### Supplementary information

Supplementary material

## Data Availability

The datasets used and/or analyzed during the current study are available from the corresponding author upon reasonable request. The original transcriptomic, proteomic, and metabonomic data have been deposited into the CNGB Sequence Archive (CNSA) of the China National GeneBank Database (CNGBdb) with accession numbers CNP0002843 and CNP0003300.
