# Supplementary material for: Ketohexokinase-dependent metabolism of cerebral endogenous fructose in microglia drives diabetes-associated cognitive dysfunction
Source: Exp Mol Med. 2023 Nov 1;55(11):2417–32. doi: 10.1038/s12276-023-01112-y (PMC10689812; doi:10.1038/s12276-023-01112-y)
Supplement: Supplementary file 1 — Supplementary material [file 12276_2023_1112_MOESM1_ESM.pdf]

Text summary

The text content：

Supplementary Material and Methods: the details of material and methods

Supplementary Table 1: the list of reagents and software and omics data

Supplementary Figure legends: Supplementary Figure Legends 1-16

Supplementary Material and Methods

1. Morris water maze (MWM)

Morris water maze is a thermostatic swimming pool with the temperature of 20-25°C. Four different shapes (circle, square, diamond and triangle) are pasted on the four directions around the maze. The hidden platform is 0.5 cm lower than the water surface in the fourth quadrant. During the 5 training days, mice were gently left into water from four-marked directions. The escape latency to the hidden platform and swimming velocity were recorded by WMT-100 Morris system (smart v3.0). On the last day, the hidden platform was removed and the number of platform crossover were observed as the criteria to evaluate the learning and memory ability. However, the swimming speed of db/db mice slowed significantly, and the path efficiency was calculated by dividing the straight distance between the entry point and the hidden platform by the total swimming pathway,[1] which balanced the side-effects of slow swimming velocity in obesity mice and is recommended to evaluate the spatial memory.

2. RNA-seq

RNA-seq was performed on 5 biological replicates for db/db, m/m mice and 6 biological replicates for BV2 cell, BV2 cell + fructose and BV2 cell + fructose +*Khk*-siRNA group. Collected hippocampus and cells were flash frozen in liquid nitrogen and stored at −80 °C. Total RNA was isolated and purified with Trizol reagent (Invitrogen, Carlsbad, CA, USA). The total RNA quantity and purity were analyzed by Bioanalyzer 2100 and RNA 1000 Nano LabChip Kit (Agilent, CA, USA) with RIN number >7.0. Library preparation was carried out with Illumina TruSeq Stranded Total RNA Library Prep Gold kit. At last, the paired-end sequencing

(PE150), was performed on Illumina Novaseq™ 6000 following the protocol recommended by the vendor.

First, cutadapt-1.9[2] was used to remove the reads containing adapter contamination, low quality bases and undetermined bases, and sequence quality was verified using FastQC v0.10.1.[3] Then hisat2-2.0.4 was used[4] to map clean reads to the genome of Homo sapiens (Ensembl v96). Stringtie-1.3.4[5] and ballgown were used to perform expression level for genes by calculating FPKM.[6] The Fold change of differentially expressed genes was > 1.2 or < 0.83, *P* value < 0.05 was statistically significance by DESeq2 screening (www.bioconductor.org/packages/release/bioc/html/DESeq2.html).[5]

3. TMT proteomics

Five separate biological replicates were performed for both db/db and m/m to quantify protein expression. The proteins were separated on 12.5% SDS-PAGE gel. Protein bands were visualized by Coomassie Blue R-250 staining. 100 μg peptide mixture per sample was labeled using TMT reagent according to the manufacturer's instructions (Thermo Fisher Scientific). TMT labeled peptides were fractionated by RP chromatography using the Agilent 1260 infinity II HPLC. The peptide mixture was diluted with buffer A (10mM HCOONH4, 5% ACN, pH 10.0) and loaded onto a XBridge Peptide BEH C18 Column, 130Å, 5 μm, 4.6 mm X 100 mm column. The peptides were eluted at a flow rate of 1 ml/min with a gradient of 0%-7% buffer B (10mM HCOONH4, 85% ACN, pH 10.0) for 5 min, 7-40% buffer B during 5-40 min, 40%–100% buffer B during 45-50 min, 100% buffer B during 50-65 min. The peptide mixture was loaded onto the C18-reversed phase analytical column (Thermo Fisher Scientific，Acclaim PepMap RSLC 50um X 15cm, nano viper, P/N164943) in buffer

A (0.1% Formic acid) and separated with a linear gradient of buffer B (80% acetonitrile and 0.1% Formic acid) at a flow rate of 300 nl/min. LC-MS/MS analysis was performed on a Q Exactive Plus mass spectrometer (Thermo Fisher Scientific) coupled to Easy nLC (Thermo Fisher Scientific) for 90 min. MS data was acquired with a data-dependent top10 method dynamically choosing the most abundant precursor ions for HCD fragmentation from the survey scan (350–1800 m/z). MS2 scans were acquired at a resolution of 17500 for HCD spectra at m/z 200 with an AGC target of $2e^5$ and a maxIT of 45 ms, and isolation width was 2 m/z. MS/MS raw files were processed using MASCOT engine (Matrix Science, London, UK; version 2.6) embedded into Proteome Discoverer 2.2, and searched against the Uniprot_MusMusculus_17027_20200226, downloaded on http://www.uniprot.org. Search parameters included trypsin, as trypsin is the enzyme used to generate peptides that allow up to 2 missed cleavages. Except for TMT labels, carbamidomethyl (C) was set as a fixed modification. Variable modifications were Oxidation(M) and Acetyl (Protein N-term). Proteins with fold change > 1.2 or < 0.83 and *P* value (Student's t test) < 0.05 were considered to be differentially expressed proteins.

4. Widely targeted metabolomics

4.1 Sample preparation and extraction

Six separate biological replicates were performed for both db/db and m/m. Metabolite extracts were prepared by adding 3 volumes of ice-cold methanol to 1 volume of plasma/serum, taking 50 mg of one tissue sample and homogenizing it with 1000 μl of ice-cold methanol/water (70%, v/v).

4.2 HPLC conditions

The sample extracts were analyzed using an LC-ESI-MS/MS system (UPLC,

Shim-pack UFLC SHIMADZU CBM A system, https://www.shimadzu.com/; MS, QTRAP® System, https://sciex.com/).

4.3 ESI-Q TRAP-MS/MS

LIT and triple quadrupole (QQQ) scans were acquired on a triple quadrupole-linear ion trap mass spectrometer (QTRAP), QTRAP® LC-MS/MS System, equipped with an ESI Turbo Ion-Spray interface, operating in positive and negative ion mode, controlled by Analyst 1.6.3 software (Sciex). Subsequent analyses were performed using MetaboAnalyst 5.0[7] (https://www.metaboanalyst.ca/home.xhtml). Differentially expressed metabolites were identified by fold change > 1.2 or < 0.83, VIP > 1, and *P* value (Student's t test) < 0.05.

5. Bioinformatics and statistics for OMICs data

5.1 Comprehensive transcriptomics and metabolomics data analysis

The comprehensive analysis of transcriptomics and metabolomics datasets and enrichment analysis of metabolomics data were performed using the "joint pathway analysis" function of MetaboAnalyst 5.0 (https://www.metaboanalyst.ca/home.xhtml) as previously described.[8] Integrated metabolic pathways include pathways containing both metabolites and metabolic genes. The hypergeometric test was used for the enrichment analysis, as the topology was measured with "degree centrality".

5.2 Gene/Protein set enrichment analysis

We performed gene set enrichment analysis using software GSEA (v4.1.0) and MSigDB to identify whether a set of genes in specific GO terms and KEGG pathways shows significant differences in two groups. Briefly, we input gene expression matrix and rank genes by Signal2Noise normalization method. Enrichment scores and *P*

value was calculated in default parameters. GO terms and KEGG pathways meeting this condition with |NES|>1, NOM *P*-val<0.05, FDR *q*-val<0.25 were considered to be different in two groups.

5.3 Correlation analysis of DEGs or DEGs and different metabolomics

Pearson's correlation analysis was performed using the OmicStudio tools at https://www.omicstudio.cn/tool, $P < 0.05$ was considered to be significantly correlated. A clustering correlation heatmap containing all *Khk* related genes was designed with OmicStudio tools (https://www.omicstudio.cn/tool). Correlation Network was performed using OmicStudio tools at https://www.omicstudio.cn/tool for all *Khk* related genes with |correlation coefficients| > 0.8.

5.4 Functional enrichment analysis

Enrichment analysis of Gene Ontology (GO) and Kyoto Encyclopedia of Genes and Genomes (KEGG) was performed with R package clusterProfiler at https://www.omicstudio.cn/tool. The GO system mainly revealed the biological processes (BP), cellular components (CC), and molecular functions (MF) involved in target genes. The focus of KEGG analysis is to explore the potential pathways in which the target genes might be involved. Enriched GO terms and KEGG pathways were determined according to the $P < 0.05$.

5.5 Volcano plot and venn diagram

The volcano plot and venn diagrams were performed at https://www.omicstudio.cn/tool.

5.6 Single cell RNA-Seq data acquisition and analysis

Using R package Seurat (4.3.0) from GEO database (http://www.ncbi.nlm.nih.gov/geo) in the calculation and analysis data set GSE201644[9 10]. In order to perform initial quality control on the extracted gene cell matrix, we screened cells with parameters of 200 ≤ number of feature RNA ≤ 5,000 and mitochondrial genes ≤ 2 0%. The "NormalizeData" and "ScaleData" functions were used to normalize and scale the gene expression matrix to obtain a linear transformation of the remaining high-quality cells. For PCA, the first 2000 variable genes were selected and the 20 most important principal components were used for cluster analysis. Subsequently, the batch effect was eliminated by combining single-cell data from multiple samples using the Harmony package's "Runharmony" feature[11]. Display clusters using uniform manifold approximation and projection (UMAP). Annotate cell types based on the expression of known markers, Such as Astrocyte (Gfap, Aqp4, Mfge8, Aldh1l1 and Aldoc), Microglia (P2ry12, Tmem119, Tnf, Gpr84, Aif1 and Hexb) and Oligodendrocyte (Fa2h, Olig1, Mog and Plp1), Endothelial (Cldn5, Flt1, Esam, Ly6c1 and Itm2a), VSMC (Rgs5, Vtn Myl9, Col4a1 and Acta2) and Ependymal (Ccdc153, Foxj1, Ttr, Enpp2, Rarres 2 and Ecrg4), Mo/Mp (Cd68, Dab2 F13a1, Lyve1, Mgl2 and Mrc1) and B cells (Cd19, Cd79a, Ms4a1 and Vpreb3), T cell (Cd3d,Cd3e and Cd3g), Neuron (Meg3, Tubb3, Stmn2, Map2 and Rbfox3), Neutrophil (Retnlg, S100a8 and S100a9), Neural stem cell (Pclaf, Sox11, Hmgb2 and Top2a) and Erythrocyte (Hbb−bt, Hba−a1 and Hba−a2).

6. Virus vectors and infections

Recombinant adeno-associated virus (rAAV2/6m) expressing U6-driven shRNA and CMV-driven EGFP (rAAV-U6-shRNA-CMV-EGFP-pA) was used. The scrambled

shRNA sequence was CCTAAGGTTAAGTCGCCCTCG, which was not targeted in both human and mouse. The KHK shRNA sequence was GCAGCGGATAGAGGAGCACAA, which was targeted mouse *Khk* (GenBank: NM_001310524.1). All AAVs were from BrainVTA Wuhan China.

At Week 20, 4 weeks prior to MWM test, mice were injected AAV. They were anaesthetized with isoflurane, and placed on stereotaxic apparatus with lidocaine subcutaneously injected for pain relief. Erythromycin eye ointment was used to prevent conjunctival infection. AAV (500 nl) was infused into the bilateral hippocampus of the brain (AP = 1.5mm posterior to bregma, ML = 1.0mm lateral to bregma, DV = 1.55mm below the skull surface) using a 5μl syringe and a 33-gauge metal needle (65460-02, Hamilton, USA), followed by a slow withdrawal over 10 min to prevent back flow. After injection, water intake and body weight of each mouse were monitored every 2 days, where the water intake was calculated from the volum difference of water remaining in the cage.

7. Western blotting

Protein concentrations were extracted from hippocampus and cells using ice-cold RIPA buffer (Beyotime, Nantong, China) containing protease inhibitor and phosphatase inhibitor (Thermo Fisher Scientific, Waltham, MA, USA). Then, the homogenate sample was centrifuged at 12000 r/min for 15 mins at 4°C, and the supernatant was collected and measured using a BCA protein assay kit (Beyotime, Nantong, China). Protein samples were dissolved in loading buffer and denatured at 99 °C for 10 mins. A 20-30 μg protein was subjected to 10%-15% SDS polyacrylamide gel electrophoresis and transferred onto polyvinylidene difluoride membranes (PVDF, Millipore, Bedford, MA, USA). The remaining protein samples

were stored at -80℃. The PVDF membranes were blocked in 5% skim milk for 1h and then incubated overnight at 4℃ with corresponding primary antibodies. Then PVDF membranes were washed with TBST membrane and incubated with appropriate secondary antibodies for 2 hrs. Specific bands of target proteins were visualized using the chemiluminescence reagents provided by ECL kit (Affinity, Shanghai, CN). The band densities were determined using Image J software (NIH, Maryland, USA) and normalized to each internal control. The list of antibodies involved in the western blot is shown in Supplementary Table 1.

8. Immunofluorescence staining and analysis

After gradient dehydration with 20% glucose and 30% glucose, mouse brain were embedded with OCT glue and frozen in a cryostat (Cryostar NX50™, Thermo Scientific) for more than 1h. The frozen brain samples were prepared into 15-μm thick sections. BV2 cells or primary microglia were seeded in 24-well plates with cell climbing slices for immunofluorescence. The tissues and cells were fixed in 4% paraformaldehyde and washed with PBS and then incubated with goat serum containing 0.2% Trion-100 for 30min at 37℃. They were intubated with primary antibodies at the 4℃ overnight and then washed with PBS (phosphate-buffered saline) for 5 min × 3 times. Fluorescent secondary antibodies were added at room temperature for 2h. Finally, DAPI (D9564, sigma, Saint Louis, USA) was added for 5min. The images were captured as soon as possible by a confocal microscope (Leica TCS SP8 STED 3X, Wetzlar, Germany). Specifically, images of spine puncta were captured by a Leica TCS SP8 DLS microscopy (Wetzlar, Germany). Imaris software (v.9.7, Bitplane, Zurich, Switzerland) was used to reconstruct and analyze immunofluorescent colocalization and microglia morphology. The antibody used for

immunofluorescence staining are listed in Supplementary Table 1.

9. Golgi staining and analysis

Golgi staining was performed as previously described with minor modifications.[12] Briefly, the brain tissue was removed and immersed in Golgi-Cox solution (consisting of 5% potassium chromate, 5% potassium dichromate, and 5% mercuric chloride) for further fixation, after which the tissue was maintained in the dark at room temperature for 2-3 days. The brains were then transferred to fresh Golgi-Cox Solution for an additional 7 days and serially sliced into 150 μm sections. The sections were washed twice in deionized water for 5 min, placed in 50% NH_4OH for 5 min, and washed again in deionized water for 5 min twice. After that, the sections were incubated in 5% sodium thiosulfate for 10 min, rinsed in PBS and then dehydrated in graded ethanol solution. The sections were observed under the bright field of a confocal microscope (Leica TCS SP8 STED 3X, Wetzlar, Germany). At an excitation wavelength of 405 nm, images were taken by z-stack scanning and the virtual color was converted into green. Imaris software (v.9.7, Bitplane, Zurich, Switzerland) were used to reconstruct and analyze neuronal dendritic arbors and dendritic spines.

10. Brain slice patch-clamp recording (Brain slice preparation)

Slice preparation was performed as described previously [12]. Briefly, 3 weeks after the bilateral hippocampal injection of AAV virus, mice were anesthetized with isoflurane and transcardially perfused with ice-cold oxygenated (95% O_2, 5% CO_2) cutting solution (95 mM NaCl, 1.8 mM KCl, 1.2 mM KH_2PO_4, 0.5 mM $CaCl_2$, 7 mM $MgSO_4$, 26 mM $NaHCO_3$, 15 mM glucose, 50 mM sucrose, pH 7.4). The mice were rapidly decapitated and their brains were removed quickly and placed in the cutting solution.

Hippocampal slices of 300 µm thickness were prepared with VT1200S Vibratome (Leica, Wetzlar, Germany), stored in an recording solution at 32 ± 1°C for 30 min, and then left at room temperature for one hour for recording. Standard whole-cell patch clamp recordings were performed in hippocampal CA1 pyramidal neurons in the same recording solution as the cutting solution, with the following exceptions: 127 mM NaCl, 2.4 mM $CaCl_2$, 1.3 mM $MgSO_4$, and 0 mM sucrose. The pipette solution for electrophysiological recording contained 125 mM K-gluconate, 5 mM KCl, 10 mM HEPES, 0.2 mM EGTA, 1 mM $MgCl_2$, 4 mM Mg-ATP, 0.3 mM Na-GTP and 10 mM phosphocreatine (pH 7.35, 290 mOsm). Data were acquired using the MultiClamp 700B amplifier (Molecular Devices, USA) and the 1550A digitizer (Molecular Devices, USA). Series resistance was monitored throughout the experiments and the cell resistance included in analysis was < 20 MΩ. Neurons would be rejected if membrane potentials were more positive than -60 mV, the Rin to Rs ratio < 5 or if series resistance fluctuated > 20% of initial values. Data were filtered at 3 kHz and sampled at 10 kHz.

11. Isolation, culture and treatment of primary microglia.

Primary mice microglia were isolated from cerebral cortices of P1 to P3 *Khk* KO or wide type mice as previously described[13]. After seeding into poly-l-lysine-coated glass coverslips, the microglia were cultured with DMEM (Gibco, Carlsbad, USA) containing 10% FBS (Sigma, California, USA) and 1% penicillin-streptomycin solution (PS, Gibco, Carlsbad, USA). The medium was exchanged every 3 days. After about 6-7 days, microglia were treated with or without 0.25mM fructose for western blotting, Immunofluorescence staining and ROS examine. To obtain the condition medium, after 24 h of fructose stimulation with or without inhibitors (GKT137831,

Mito-Tempo and N-acetylcysteine), all medium were changed to a fresh neurobasal for another 24 h. The conditioned neurobasal medium was obtained and centrifuged at 1500 rpm for 10 min. The supernatant was collected, stored at -20 °C, and treated primary neurons according to the experiment.

12. BV2 Cell Culture and treatment.

BV2 microglial cell line was maintained in high glucose DMEM containing 10% (v/v) FBS (Gemini, Cleveland, USA) and 1% PS (Gibco, Carlsbad, USA) at 37°C in a humidified environment of 95% air and 5% CO_2, with medium changing every 2 days. Cells were cultured to 75% confluence in 6-well plates for 24 h experiments involving insult with fructose (MCE, Monmouth Junction, USA) or osthole (Santa cruz, Santa cruz, USA) at different concentration.

Khk-siRNA, negative control siRNA (NC-siRNA) and siRNA-Mate Transfection Reagent for siRNA transfection were purchased from GenePharma Co., Ltd. (Shanghai, China). The sequences for mouse *Khk* siRNAs are designed as follows: 5′-GGUGGUGUUUGUCAGCAAATT-3′, 5′- CCCGUACCAUUAUACUCUATT-3′, 5′-AGCGGAUAGAGGAGCACAATT-3′. The negative control siRNA contains a scrambled sequence that will not result in degradation of cellular mRNA. BV2 cells were transfected with siRNA-siRNA-Mate complex for 6 h according to the manufacturer’s protocols. After 48-72 h, they were treated with fructose.

13. Isolation, culture and treatment of primary neuron.

Primary cultures of hippocampal neurons were isolated from prenatal mice as previous described[14]. Neurons were cultured in Neurobasal (Thermo Fisher Scientific, Waltham, MA, USA) with 2% B27 (Thermo Fisher Scientific, Waltham, MA, USA),

2mM L-glutamine (Gibco, Carlsbad, USA) and 1% PS for 7-9 days. After treated with conditioned neurobasal medium (supplemented with 2% B27, 2mM L-glutamine and 1% PS) for 24 h, protein was extracted for western blotting.

14. HT22 cell culture and treatment

Immortalized mouse HT22 hippocampal neurons (Beina Chuanglian Biotech Institute, Beijing, China) were cultured in DMEM supplemented with 10% FBS, 1% PS at 37 °C in 5% CO_2. The DMEM was changed every 1-2 days. Protein was extracted under different BV2 cell condition medium treating for 24 h.

Supplementary Table 1. List of reagents, software and data

Antibodies	Source	Identifier	Use
Mouse monoclonal<br>anti-Aldose Reductase	Santa Cruz<br>Biotechnology	sc-166918	WB: 1:400
Mouse monoclonal<br>anti-Glut5	Santa Cruz<br>Biotechnology	sc-271055	WB: 1:50<br>IF: 1:50
Mouse monoclonal<br>anti-Ketohexokinase	Santa Cruz<br>Biotechnology	sc-377411	WB: 1:50<br>IF: 1:25
Chicken polyclonal<br>anti-GFAP	Genetex	GTX85454	IF: 1: 300
Mouse monoclonal<br>anti-Iba1	Santa Cruz	sc-32725	IF: 1: 25
Rabbit polyclonal<br>anti-Iba1	Wako	019-19741	IF: 1: 500
Rabbit monoclonal<br>Anti-NeuN	Abcam	ab177487	IF: 1: 100
Mouse monoclonal<br>anti-Gp91-phox	Santa Cruz<br>Biotechnology	sc-130543	WB:1: 500
Mouse monoclonal<br>anti-P22-phox	Santa Cruz<br>Biotechnology	sc-271968	WB:1: 100
Rabbit Polyclonal<br>anti-NOX4	Proteintech	14347-1-AP	WB: 1:4000<br>IF: 1:25
Mouse Monoclonal<br>anti-NOX4	Santa Cruz<br>Biotechnology	sc-518092	IF: 1:25
Rabbit monoclonal	Abcam	ab238135	WB: 1:1000

Anti-PSD95			IF: 1:25
Rabbit monoclonal anti-Synaptophysin	Abcam	ab32127	WB: 1:10000
Alexa Fluor 594 anti-synaptophysin [YE269]	Abcam	ab206868	IF: 1:50
NDUFS8	Proteintech	25172-1-AP	WB: 1:1000
SDHB	Genetex	GTX113833	WB: 1:1000
CYTB	Proteintech	55090-1-AP	WB: 1:500
Complex IV	Proteintech	11242-1-AP	WB: 1:1000<br>IF: 1:300
ATPase IF1	Abcam	ab110277	WB: 1:1000
HRP labeled goat anti-rabbit IgG (H+L)	Diyibio	DY60202	WB:1:5000
HRP labeled goat anti-mouse IgG (H+L)	Diyibio	DY60203	WB: 1:5000
Donkey Anti-Rabbit IgG H&L (Alexa Fluor® 594)	Abcam	ab150064	IF: 1:500
Goat polyclonal Secondary Antibody to Mouse IgG - H&L (Alexa Fluor® 647)	Abcam	ab150115	IF: 1:500
Donkey polyclonal	Jackson	RRID:	IF: 1:500

Anti-Chicken IgY (IgG) (H+L)(Alexa Fluor® 488)	immunoresearch lab	AB_2340375	
Donkey anti-Mouse IgG (H+L)(Alexa Fluor 594)	Invitrogen	A-21203	IF: 1:500
Donkey polyclonal anti-Rabbit IgG (H+L)(Alexa Fluor Plus 488)	Invitrogen	A32790	IF: 1:500
Donkey polyclonal anti-rabbit IgG (H+L)(Alexa Fluor 647)	Invitrogen	A31573	IF: 1:500
**Chemicals, peptides, and recombinant proteins**			
Osthole	Santa cruz	sc-205780	KHK inhibitor
Fructose	MCE	HY-N0395	Cell treatment
TTX	MREDA	M046335	voltage-gated sodium channel blocker
Picrotoxin	Tocris	13A/141261	$GABA_A$ antagonist
GKT137831	MCE	HY-12298	NOX4 inhibitor
N-Acetylcysteine	MCE	HY-B0215	ROS scavenger
mitoTEMPO	GLPBIO	GC44206	Mitochondrial ROS inhibitor

**Commercial assays**			
Tissue Reactive Oxygen Species (ROS) Detection Assay Kit	Bestbio	BB-470512-1	Examining ROS level of hippocampus
Cell Reactive Oxygen Species (ROS) Detection Assay Kit	Beyotime	S0033S	Examining ROS level of BV2
Lipid Peroxidation MDA Assay Kit	Beyotime	S0131S	Examining MDA level
Mouse 8-Hydroxy-desoxyguanosine (8-OHdG) ELISA Kit	SinoBestBio	YX-E27780	Examining 8-OHdG level
Mouse Nitrotyrosine (NT) ELISA Kit	SinoBestBio	YX-E23780	Examining NT level
Enhanced ATP Assay Kit	Beyotime	S0027	Examining NT level
Fructose Assay Kit	Nanjing Jiancheng Bioengineering Institute	A085-1-1	Examining fructose level
Sorbitol Assay Kit	Solarbio	BC2525	Examining Sorbitol level
Mouse INS (Insulin) ELISA Kit	Xinlebio	Xl-Em0483	Examining Insulin level

Enhanced ATP Assay Kit	Beyotime	S0027	Examining ATP level
**Deposited data**			
Serum metabolomics data	This paper	CNP0002843	Bioinformatics analysis
Hippocampus metabolomics data	This paper	CNP0002843	Bioinformatics analysis
Hippocampus proteomics data	This paper	CNP0002843	Bioinformatics analysis
Hippocampus transcriptomics data	This paper	CNP0002843	Bioinformatics analysis
BV2 cell transcriptomics data	This paper	CNP0003300	Bioinformatics analysis
**Experimental models: Organisms/strains**			
db/db mouse	Model Animal Research Center of the Nanjing University.	N/A	Type 2 diabetic model
Khk KO mouse	Cyagen Biosciences	N/A	Primary microglia
**Software and algorithms**			
IMARIS software	Bitplane	https://imaris.oxinst.com/	Reconstruction and analysis of Immunofluorescence and golgi

			Staining image
GraphPad Prism 7	GraphPad	N/A	statistics
Bioinformatic analysis	Lc-bio	https://www.omicstudio.cn/tool.	Enrichment analysis; Correlation analysis; Volcano plot; GSEA analysis; Differential expression analysis
Comprehensive analysis	N/A	https://www.metaboanalyst.ca/home.xhtml	Comprehensive analysis of transcriptomics and metabolomics
ImageJ	NIH	https://imagej.nih.gov/ij/	Synaptic analysis
Mini Analysis	Synaptosoft	www.synaptosoft.com	Electrophysiology analysis
Clampfit	Molecular Devices	N/A	Electrophysiology analysis

Supplementary Figure Legends

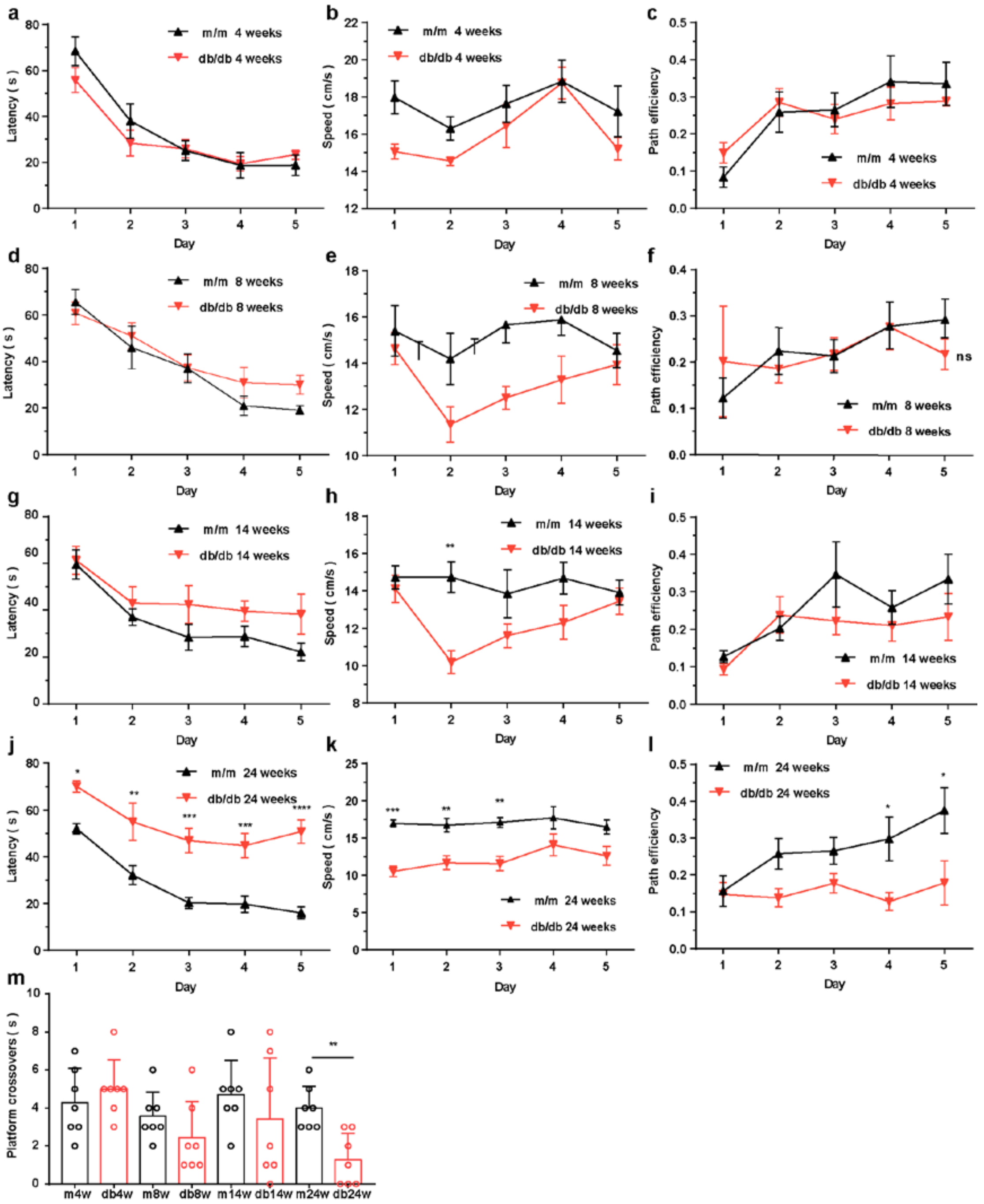

Supplementary Fig. 1. Periodic measurements of cognitive status of db/db and m/m mice.

(a-c) The escape latency (a), swimming speed (b) and path efficiency (c) of 4-week db/db and m/m mice during the training session of MWM test. (d-f) The escape latency (d), swimming speed (e) and path efficiency (f) of 8-week db/db and m/m

mice during the training session. (g-i) The escape latency (g), swimming speed (h) and path efficiency (i) of 14-week db/db and m/m mice during the training session. (j-l) The escape latency (j), swimming speed (k) and path efficiency (l) of 24-week db/db and m/m mice during the training session. (m) The number of platform crossovers in the probe trial of MWM in db/db and littermate m/m mice at the 4, 8, 14 and 24 weeks. All data were presented as the mean ± SEM. The results in (a-l) were analyzed by two-way ANOVA. The results in (m) were analyzed by one-way ANOVA with Tukey's multiple comparisons test. n = 7 mice per group. $*P < 0.05$; $**P < 0.01$; $***P < 0.001$; $****P < 0.0001$.

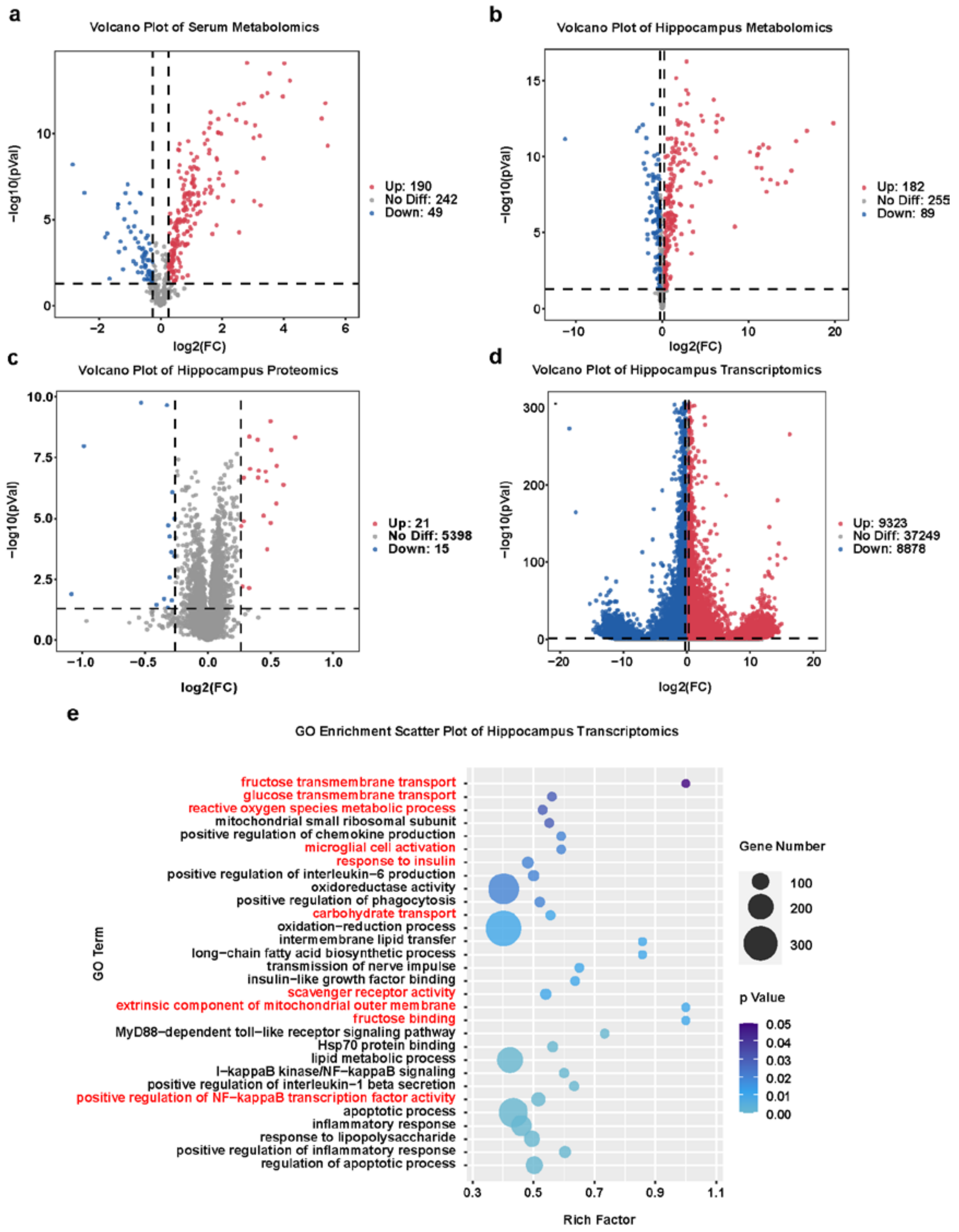

Supplementary Fig. 2. Multi-omics of serum and/or hippocampus of db/db and m/m mice.

(a, b) Volcano plot of widely targeted metabolomics for the serum (a) and hippocampus (b) exhibited the differential metabolites in the db/db vs. m/m mice (n = 6). (c, d) Volcano plot of proteomic and transcriptomic sequencing of the

hippocampus showing the differential proteins (c) and genes (d) between db/db and m/m mice (n = 5). The data in (a-d) are described by red dot (up-regulated) and blue dot (down-regulated). (e) The GO enrichment analysis of hippocampus transcriptomics.

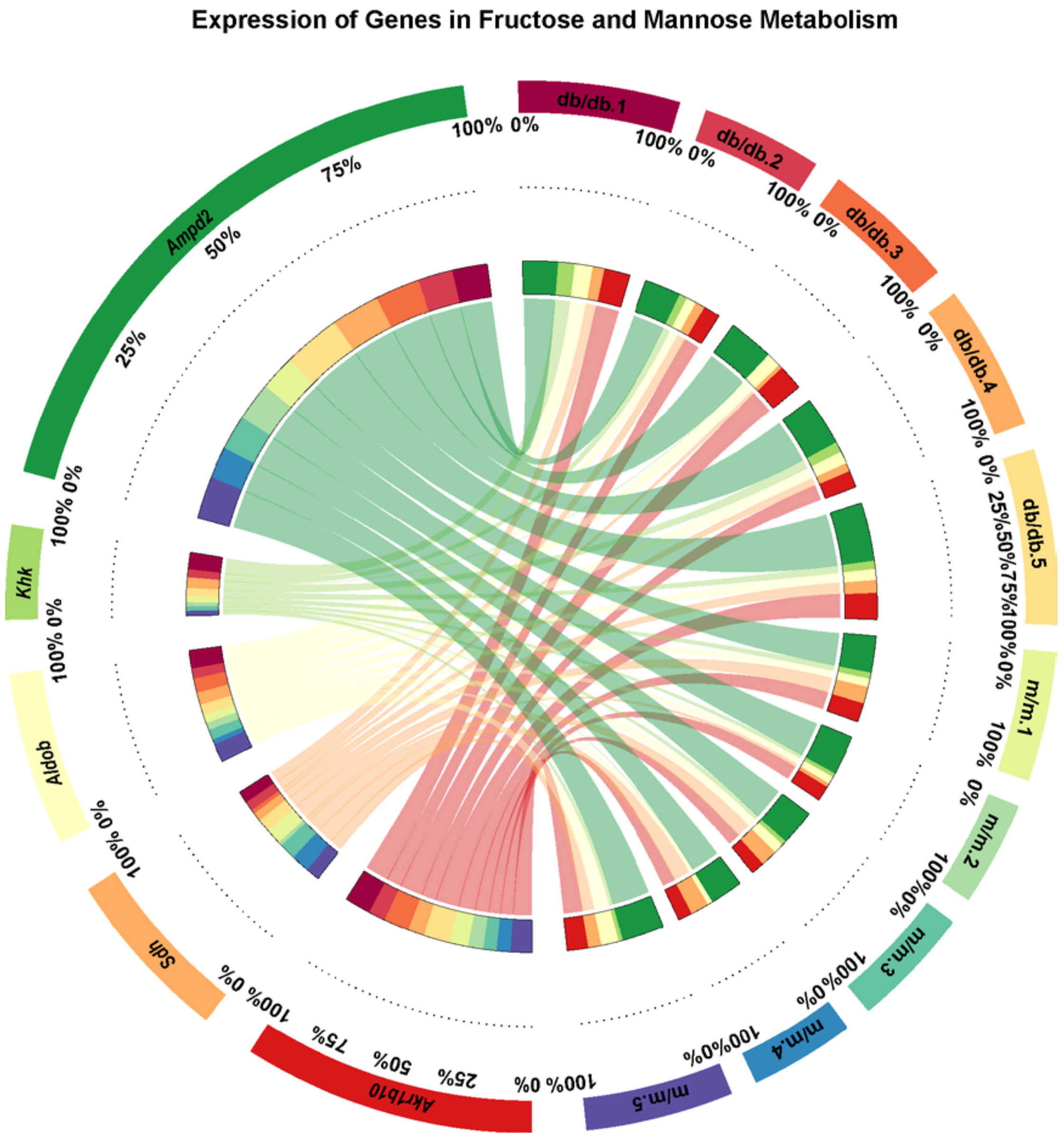

Supplementary Fig. 3. Expression pattern of DEGs involved in "fructose and mannose metabolism" pathway in RNA-seq analysis.

The left semicircle represents key genes of the "fructose and mannose metabolism" pathway in DEGs, and the length of the arc indicates the abundance; the right semicircle represents 5 separate biological replicates for each group.

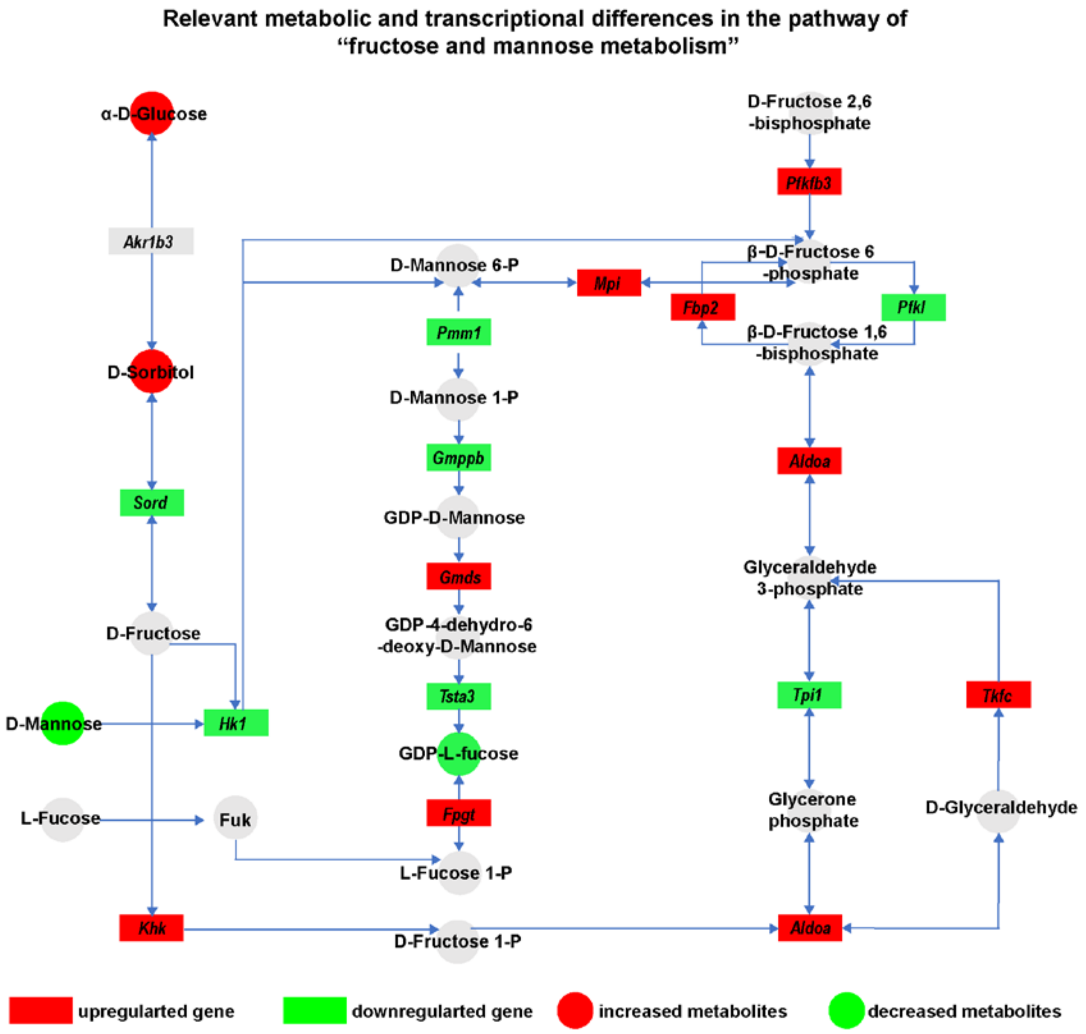

Supplementary Fig. 4. Relevant differences in the pathway of "fructose and mannose metabolism".

Schematic representation of relevant metabolic and transcriptional differences in the pathway of "fructose and mannose metabolism" by comprehensive analysis (P = 3.53 e-06). The circles present metabolites, and the rectangles present genes. The red one indicates a significant increase in metabolite concentrations or gene expression levels, while the green one indicates significantly declined concentrations of metabolites or expression levels of genes.

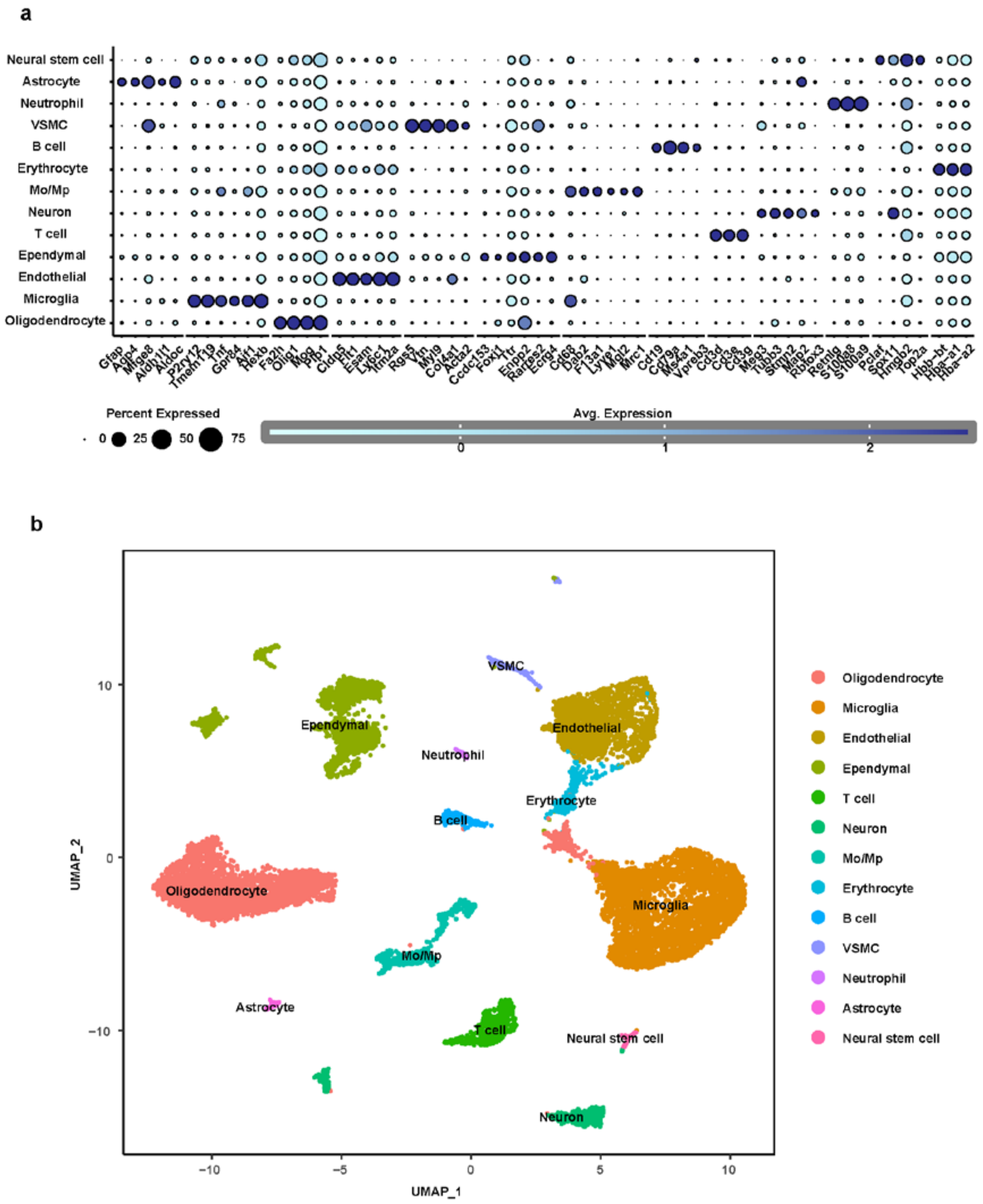

Supplementary Fig. 5. Cell annotation of scRNA-seq dataset GSE201644.

(a) Dot plot showing the specific genes for different cells. (b) Uniform Manifold Approximation and Projection (UMAP) plot of cells isolated from db/db and db/m hippocampus exhibiting 13 distinct cell types.

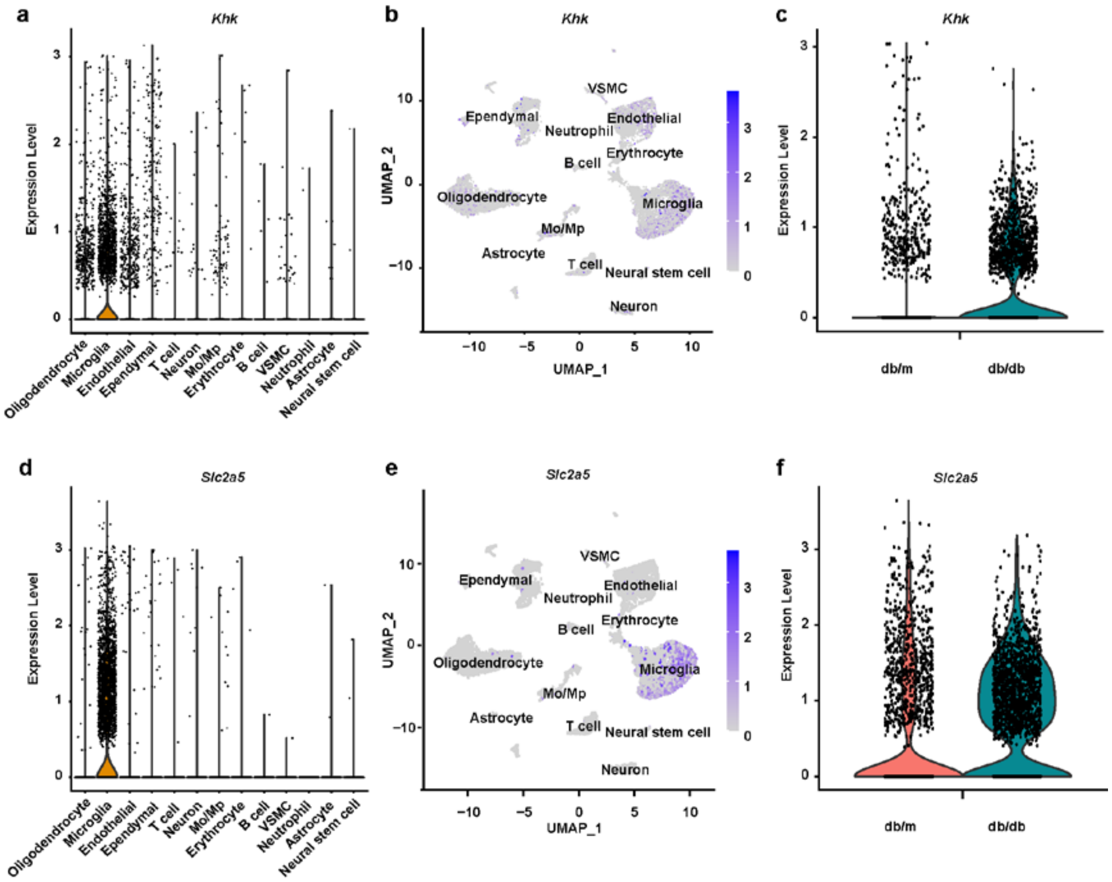

Supplementary Fig. 6. The expression of *Khk* and *Slc2a5* in microglia in dataset GSE201644.

(a) Violin plot of *Khk* expression in the annotated cells. (b) Uniform Manifold Approximation and Projection (UMAP) plot showed the expression of *Khk* in different cells. (c) Violin plot of *Khk* expression in the microglia of db/db and db/m. (d) Violin plot of *Slc2a5* expression in the annotated cells. (e) Uniform Manifold Approximation and Projection (UMAP) plot showed the expression of *Slc2a5* in different cells. (f) Violin plot of *Slc2a5* expression in the microglia of db/db and db/m.

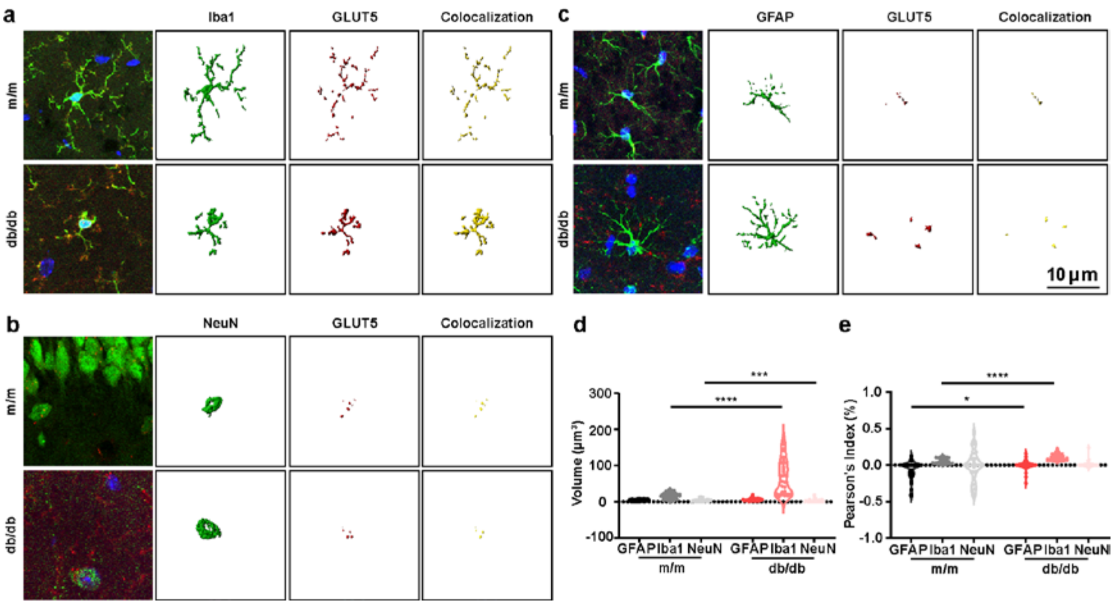

Supplementary Fig. 7. Expression and localization of GLUT5 in the hippocampus of db/db mice.

(a-c) Representative confocal images and three-dimensional reconstruction of GLUT5 (red) colocalization status in microglia (a, green), neuron (b, green) or astrocytes (c, green). DNA was labeled by DAPI (blue). Scale bar = 10 μm. (d) Volume of GLUT5 and cell markers colocalization intensity (n = 30 from 4-5 mice). (e) Correlation of GLUT5 and cell markers fluorescence intensity (n = 30 from 4-5 mice). All data were presented as the mean ± SEM and were analyzed by student's t test or Mann-Whitney test based on the results of normal distribution test. **P* < 0.05; ***P* < 0.01; ****P* < 0.001; *****P* < 0.0001.

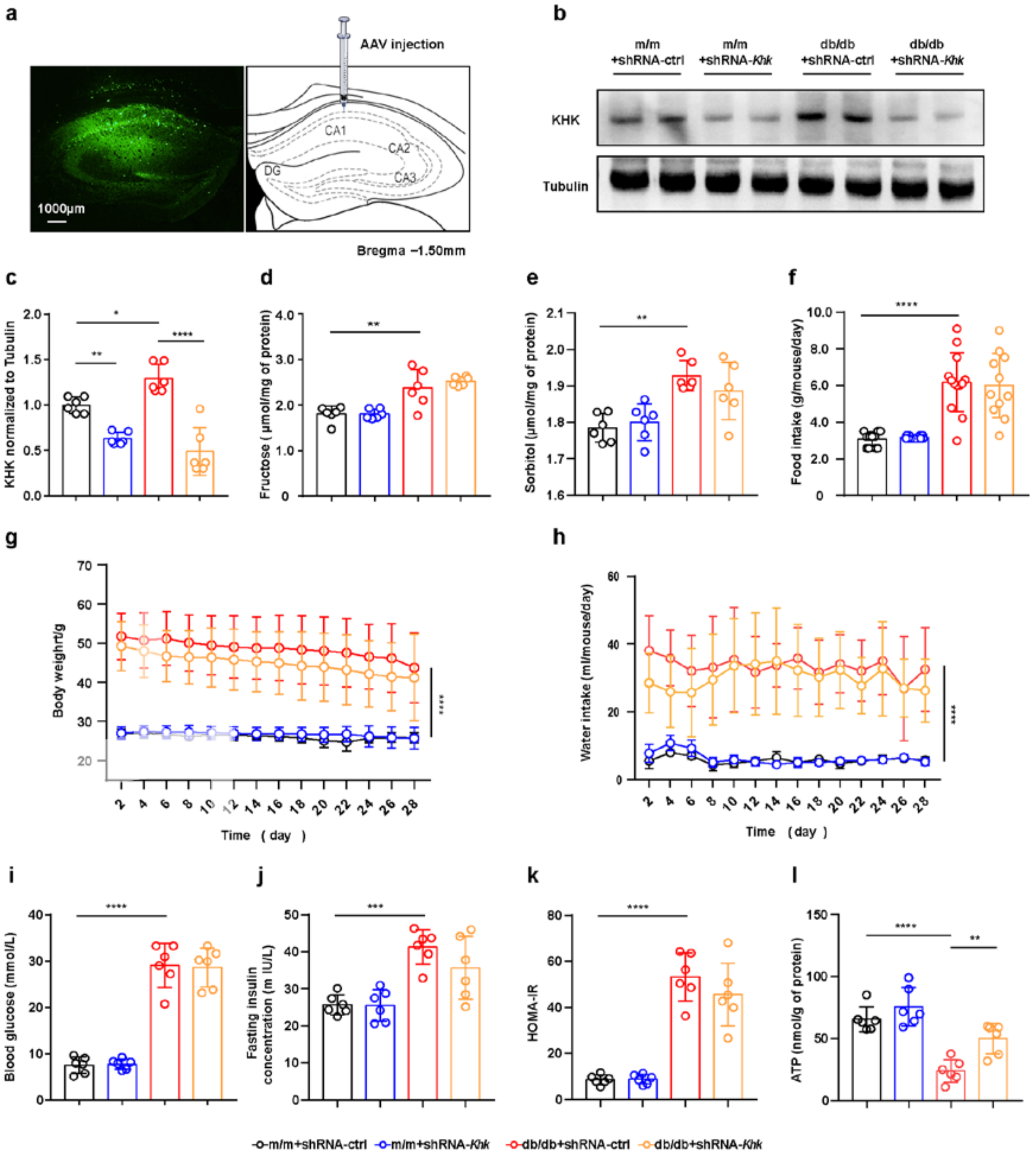

Supplementary Fig. 8. *Khk* knockdown increases ATP level in the hippocampus of db/db mice.

(a) Expression of AAV-encoded EGFP carrying *Khk*-shRNA-EGFP 3 weeks after ICV injection. (b, c) Representative western blot (b) and densitometric analysis (c) of KHK (n = 6). (d-k) The effect of *Khk* knockdown on fructose (d) and sorbitol levels (e), or physical parameters such as food intake (f), body weight (g), water intake (h), blood glucose (i), fasting insulin concentration (j), or HOMA-IR (k) (n = 6-12). (l) *Khk* knockdown increases ATP level in the hippocampus of db/db mice (n = 6). The

data were presented as mean ± SEM. The data in (c-f, i-l) were analyzed by one-way ANOVA with Tukey post hoc analysis. The data in (g, h) were analyzed by two-way ANOVA. *P < 0.05; **P < 0.01; ***P < 0.001; ****P < 0.0001.

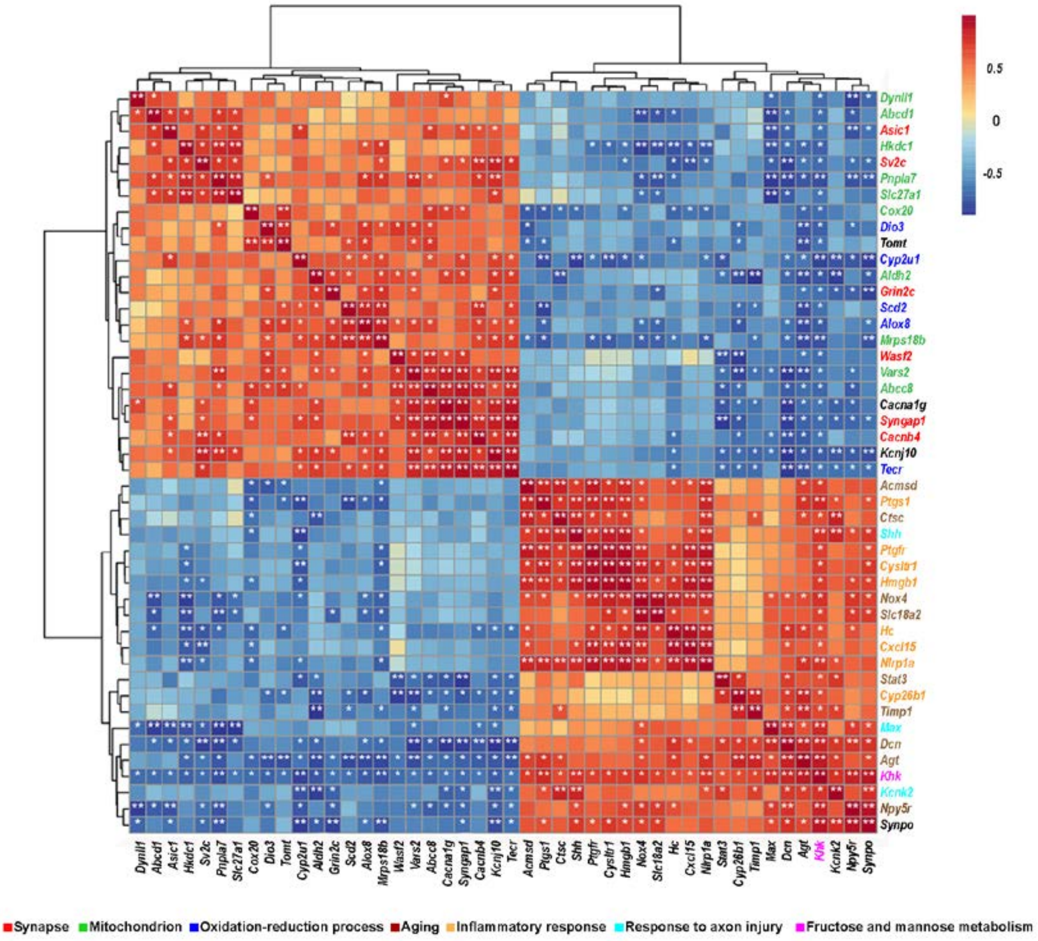

Supplementary Fig. 9. Heat map of representative DEGs correlated with *Khk*.

In RNA-seq analysis, 287 DEGs were correlated with *Khk*, 24 of which were negatively correlated and enriched in the GO terms such as “synapse”, “mitochondrion”, “oxidation-reduction process”, and the other 21 DEGs were positively correlated and enriched in “aging”, “inflammatory response” and “response to axon injury”.

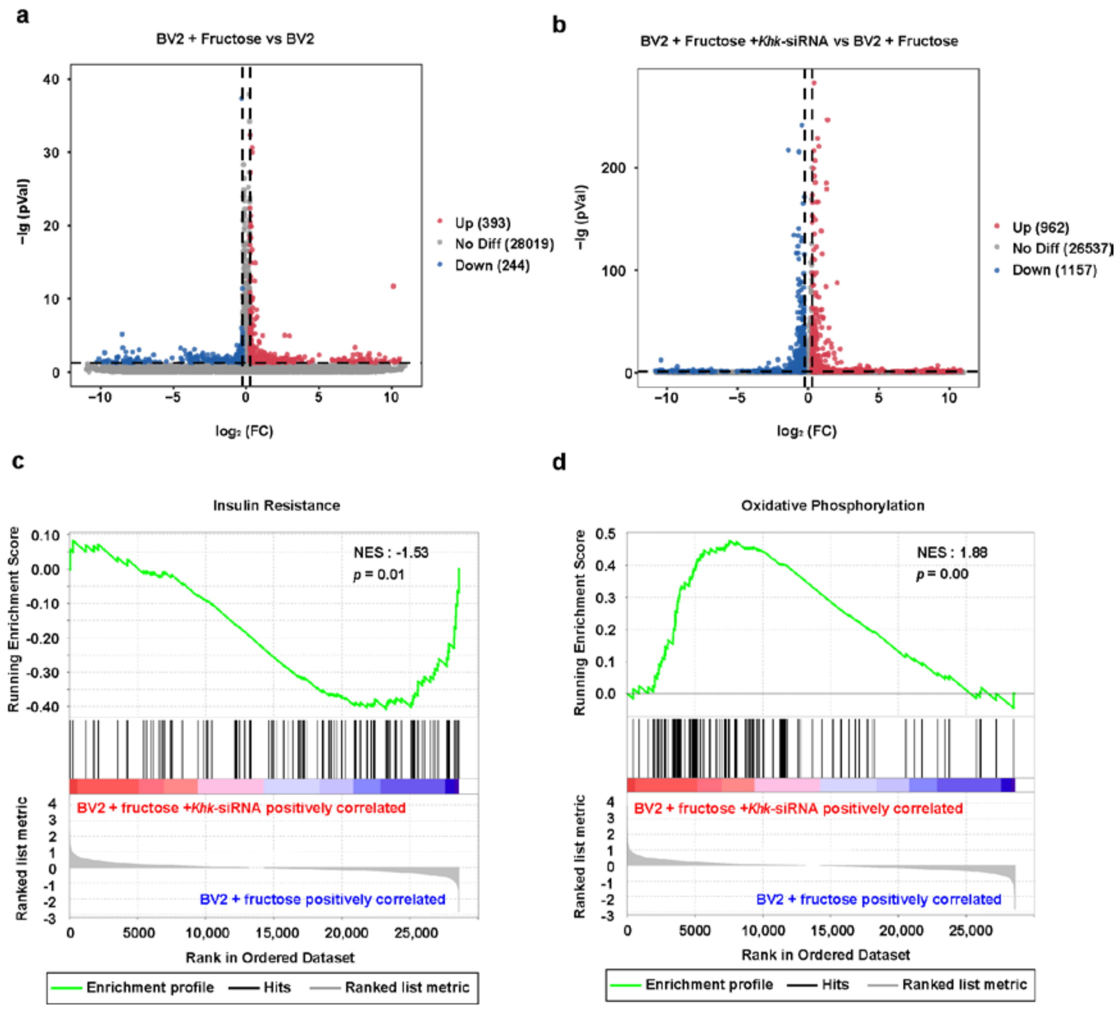

Supplementary Fig. 10. Alternations in gene expression of BV2 cells after fructose treatment or *Khk* depletion.

(a, b) Volcano plot showing upregulation (red) or downregulation (blue) in gene expression of BV2 cells in indicated conditions. (n = 6) (c, d) The alternation of "insulin resistance" pathway (c) and "oxidative phosphorylation" pathway (d) in different conditions via GSEA.

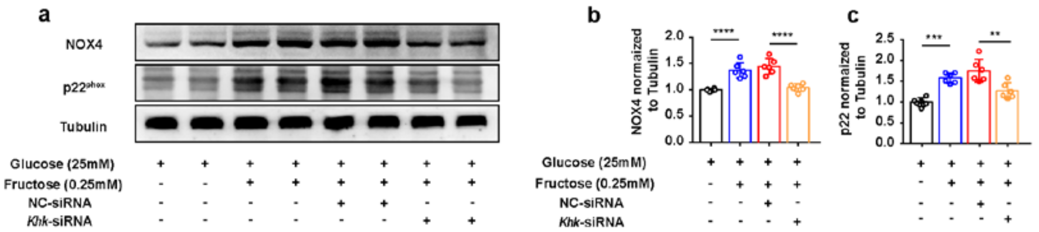

Supplementary Fig. 11. The expression of NOX4 and p22^{phox} in BV2 cells after the treatment of fructose or knocking down *Khk*.

(a) Representative western blot of NOX4 and p22^{phox} in BV2 cell under different conditions. (b,c) Densitometric analysis of NOX4 (b) and p22^{phox} (c) in BV2 cell under different conditions (n = 6).

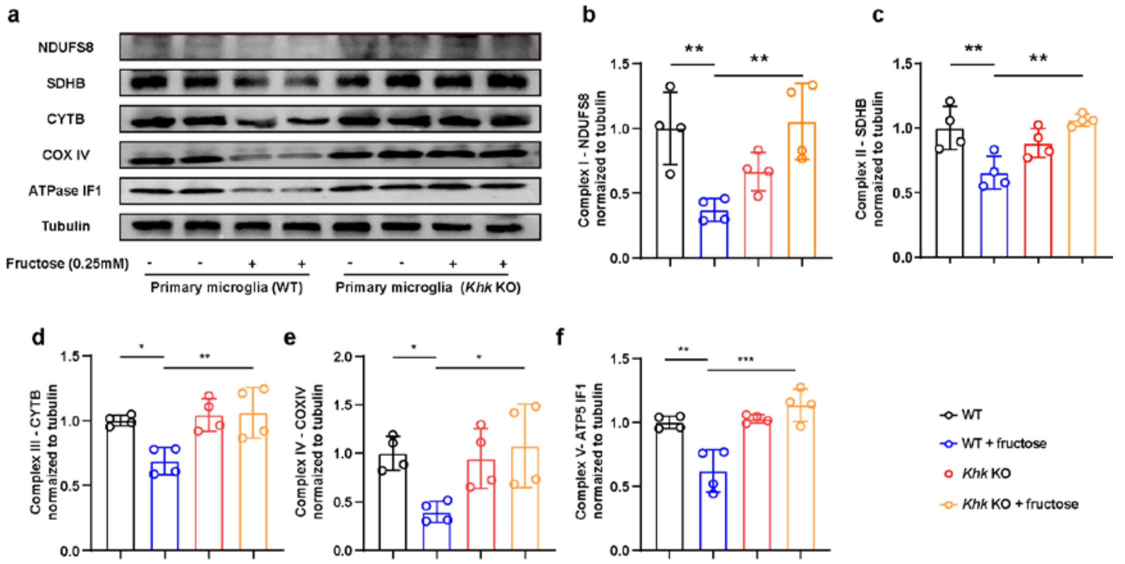

Supplementary Fig. 12. The expression of OXPHOS related proteins in WT and *Khk* KO primary microglia after the treatment of fructose.

(a) Representative western blot of OXPHOS related proteins, including NDUFS8 (complex I), SDHB (complex II), CYTB (complex III), COX IV (complex IV) and ATPase IF1 (complex V) in wild type (WT) or *Khk* KO mice primary microglia with or without fructose treatment. (b-f) Densitometric analysis of OXPHOS related proteins in wild type (WT) or *Khk* ko primary microglia with or without fructose treatment (n = 4).

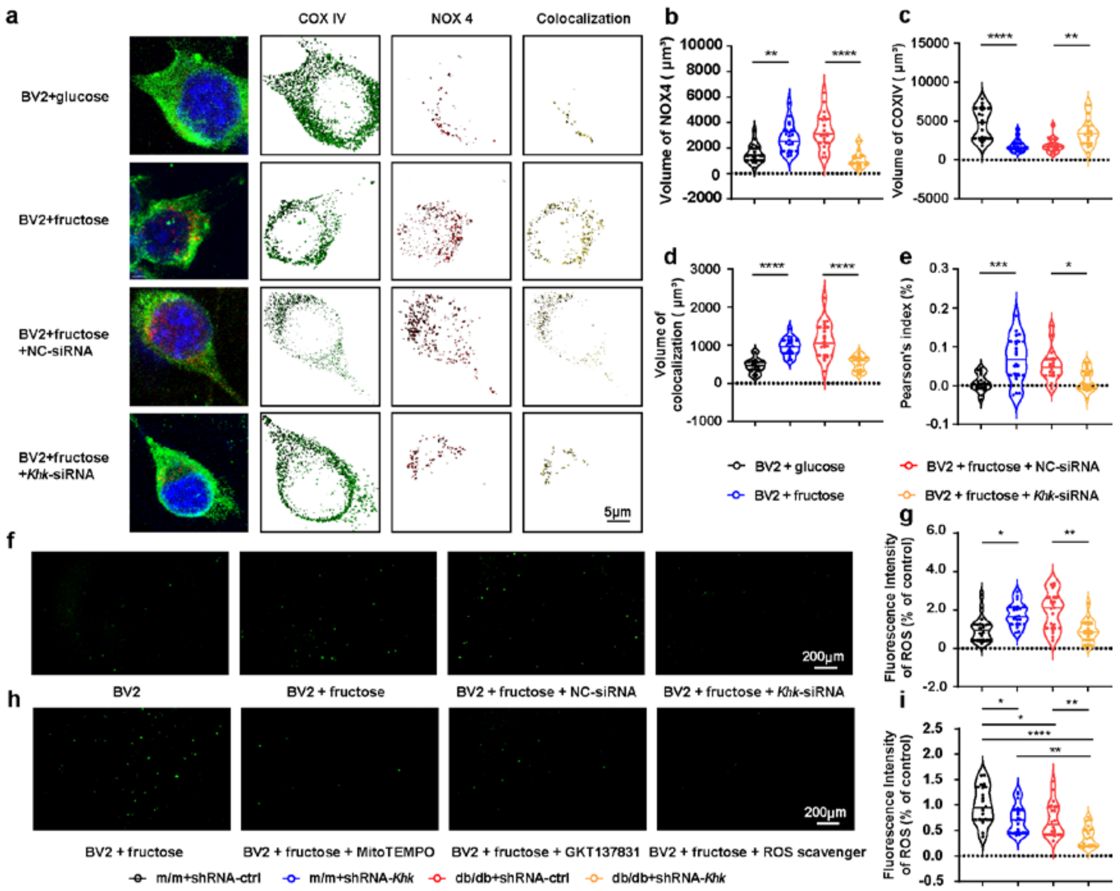

Supplementary Fig. 13. The alternations of NOX4 mitochondrial translocation and ROS production in BV2 cell treated with or without fructose or *Khk*-siRNA

(a) Representative immunofluorescence image and three-dimensional reconstruction of the mitochondrial marker NOX4 (red) and COX IV (green) in BV2 cell lines at the absence or presence of fructose or *Khk*-siRNA. DNA was labeled by DAPI (blue). Bar = 5 μm. (b-d) Fluorescence intensity of NOX4 (b), COX IV (c) and their colocalization (d) (n = 18 cells from 3 independent experiments). (e) Correlation of NOX4 and COX IV fluorescence intensity (n = 18 cells from 3 independent experiments). (f, g) Representative fluorescence images (f) and quantification analysis (g) of ROS fluorescence intensity in BV2 cells treated with or without fructose and *Khk*-siRNA (n = 18 from 3 independent experiments). Scale bar = 200 μm. (h, i)

Representative fluorescence images (h) and quantification analysis (i) of ROS fluorescence intensity of BV2 cells treated with or without NOX4 inhibitor (GKT137831), mitochondrial ROS inhibitor (Mito-Tempo) or ROS scavenger (N-acetylcysteine) in fructose-loaded medium (n = 18 from 3 independent experiments). Scale bar = 200 μm. The data were presented as mean ± SEM and were analyzed by one-way ANOVA with Tukey post hoc analysis (b, c, e, i) or Kruskal-Wallis followed by Dunn's multiple comparisons tests (d, g). $^*P < 0.05$; $^{**}P < 0.01$; $^{***}P < 0.001$; $^{****}P < 0.0001$.

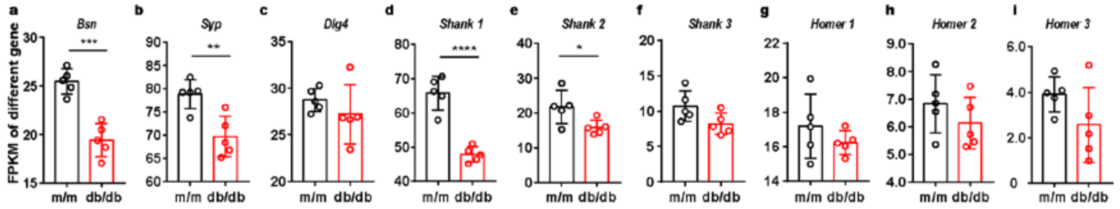

Supplementary Fig. 14. The alternations in abundance of synaptic proteins from RNA-seq of hippocampus.

The alternations in Bsn (a), Syp (b), Dlg4 (c), Shank1 (d), Shank2 (e), Shank3 (f), Homer1 (g), Homer2 (h) and Homer3 (i) from RNA-seq of hippocampus between db/db mice vs m/m mice (n = 5). The data were presented as mean ± SEM and were analyzed by Student's t test. **P* < 0.05; ***P* < 0.01; ****P* < 0.001; *****P* < 0.0001.

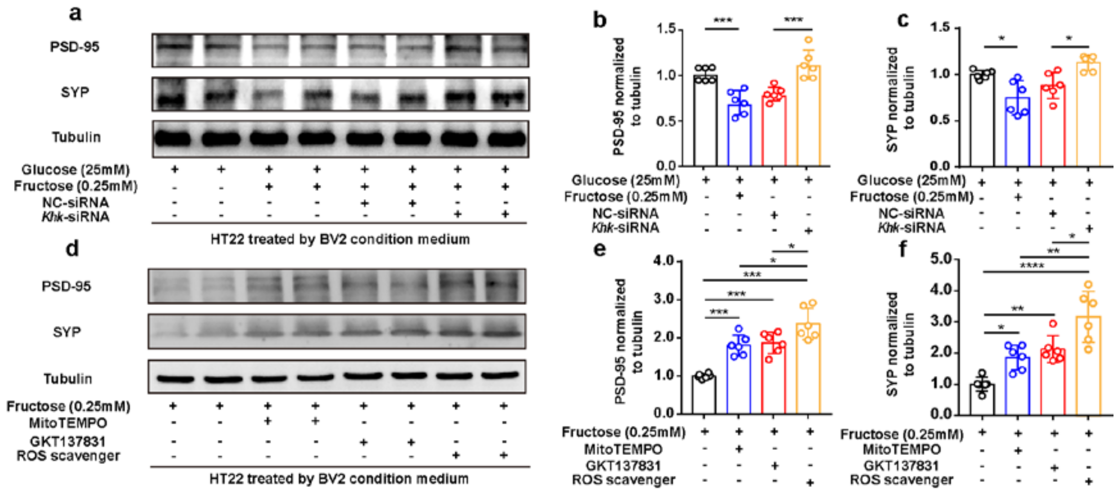

Supplementary Fig. 15. The expression of PSD-95 and SYP in HT22 cells after the treatment of BV2 condition medium.

(a-c) BV2 cells were treated with or without fructose, NC-siRNA or *Khk*-siRNA, then the BV2-conditioned medium was collected for HT22 cell culture. Representative western blot (a) and densitometric analysis of PSD-95 (b) and SYP (c) in HT22 cells (n = 6). (d-f) BV2 cells were treated with or without MitoTEMPO, GKT137831 and ROS scavenger in fructose-loaded medium and the BV2-conditioned medium were collected for HT22 cell culture. Representative western blot (d) and densitometric analysis of PSD-95 (e) and SYP (f) expression in HT22 cells (n = 6). The data were presented as mean ± SEM and were analyzed by one-way ANOVA with Tukey post hoc analysis (b, c, e, f). **P* < 0.05; ***P* < 0.01; ****P* < 0.001; *****P* < 0.0001.

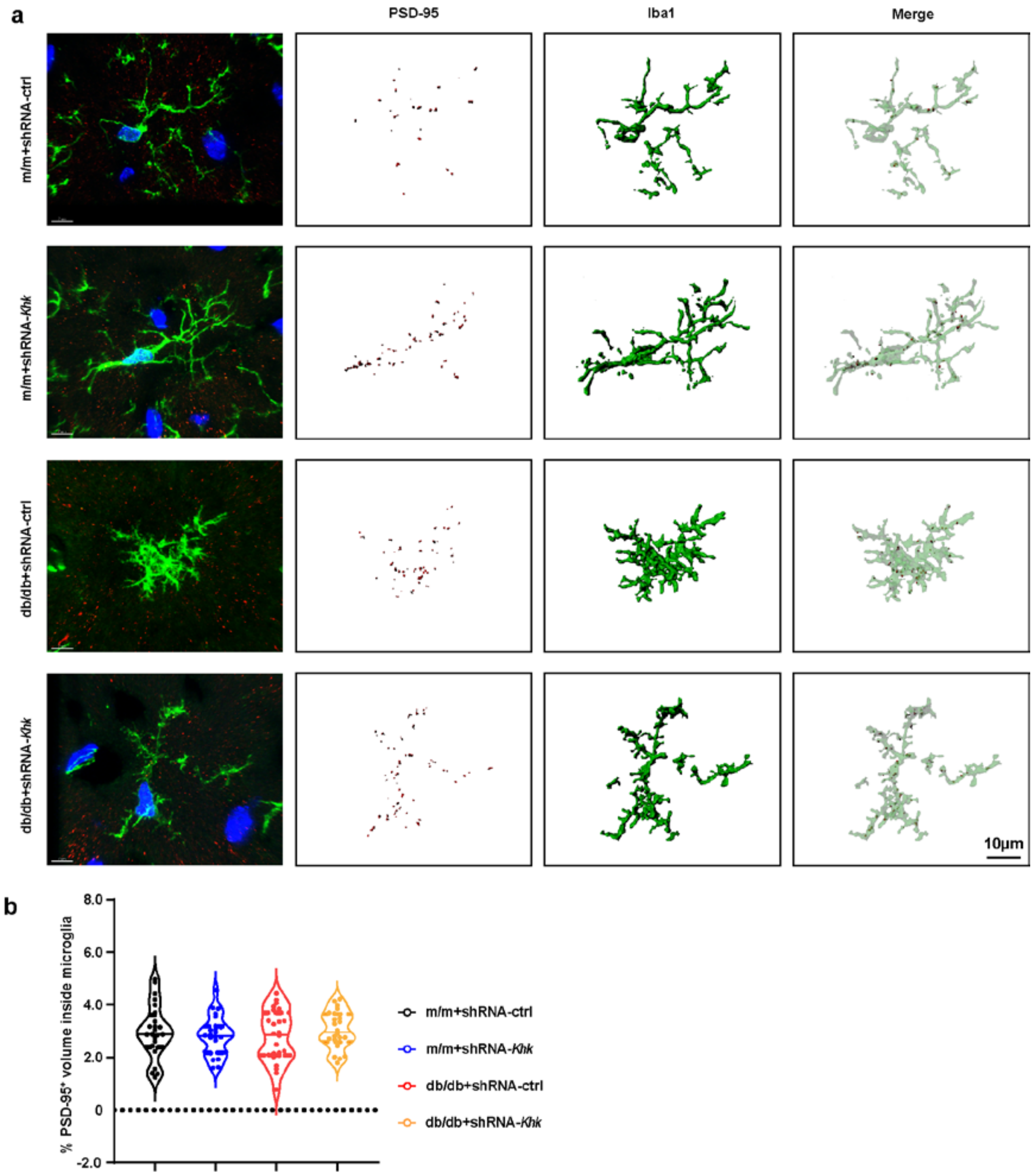

Supplementary Fig. 16. The effect of *Khk* knockdown on microglia-mediated engulfment of synapses

(a) Representative immunofluorescence image and three-dimensional reconstruction of PSD-95 puncta internalized in microglia (PSD-95/Iba1). Scale bar = 10 μm. (b) Percentage of PSD-95$^+$ volume within microglia (n = 24 from 3-4 mice). The data were presented as mean ± SEM and were analyzed by one-way ANOVA with Tukey post hoc analysis (b). **P* < 0.05; ***P* < 0.01; ****P* < 0.001; *****P* < 0.0001.

Reference

1. Ma, H. et al. Transplantation of platelet-derived mitochondria alleviates cognitive impairment and mitochondrial dysfunction in db/db mice. *Clin Sci (Lond)*. **134**, 2161-2175 (2020).
2. Kechin, A., Boyarskikh, U., Kel, A. & Filipenko, M. cutPrimers: A New Tool for Accurate Cutting of Primers from Reads of Targeted Next Generation Sequencing. *J Comput Biol*. **24**, 1138-1143 (2017).
3. Beekman, R. et al. The reference epigenome and regulatory chromatin landscape of chronic lymphocytic leukemia. *Nat Med*. **24**, 868-880 (2018).
4. Kim, D., Langmead, B. & Salzberg, S. L. HISAT: a fast spliced aligner with low memory requirements. *Nat Methods*. **12**, 357-360 (2015).
5. Pertea, M. et al. StringTie enables improved reconstruction of a transcriptome from RNA-seq reads. *Nat Biotechnol*. **33**, 290-295 (2015).
6. Trapnell, C. et al. Transcript assembly and quantification by RNA-Seq reveals unannotated transcripts and isoform switching during cell differentiation. *Nat Biotechnol*. **28**, 511-515 (2010).
7. Pang, Z. et al. MetaboAnalyst 5.0: narrowing the gap between raw spectra and functional insights. *Nucleic Acids Res*. **49**, W388-W396 (2021).
8. Mao, K. et al. An Integrative Transcriptomic and Metabolomic Study Revealed That Melatonin Plays a Protective Role in Chronic Lung Inflammation by Reducing Necroptosis. *Front Immunol*. **12**, 668002 (2021).
9. Ma, S. et al. Single-Cell Sequencing Analysis of the db/db Mouse Hippocampus Reveals Cell-Type-Specific Insights Into the Pathobiology of Diabetes-Associated Cognitive Dysfunction. *Front Endocrinol (Lausanne)*. **13**, 891039 (2022).
10. Stuart, T. et al. Comprehensive Integration of Single-Cell Data. *Cell*. **177**, 1888-1902 (2019).
11. Korsunsky, I. et al. Fast, sensitive and accurate integration of single-cell data with Harmony. *Nat Methods*. **16**, 1289-1296 (2019).
12. Li, Z.-Z. et al. Extracellular matrix protein laminin β1 regulates pain sensitivity and anxiodepression-like behaviors in mice. *J Clin Invest*. **131**, e146323 (2021).
13. Yang, J. et al. RIPK3/MLKL-Mediated Neuronal Necroptosis Modulates the M1/M2 Polarization of Microglia/Macrophages in the Ischemic Cortex. *Cereb Cortex*. **28**, 2622-2635 (2018).
14. Tang, W. et al. Caveolin-1 Alleviates Diabetes-Associated Cognitive Dysfunction Through Modulating Neuronal Ferroptosis-Mediated Mitochondrial Homeostasis. *Antioxid Redox Signal*. **37**, 867-886 (2022).
